# An integrative transcriptomic approach to identify depot differences in genes and microRNAs in adipose tissues from high fat fed mice

**DOI:** 10.18632/oncotarget.24226

**Published:** 2018-01-13

**Authors:** Nadeeja N. Wijayatunga, Mandana Pahlavani, Nishan S. Kalupahana, Kameswara Rao Kottapalli, Preethi H. Gunaratne, Cristian Coarfa, Latha Ramalingam, Naima Moustaid-Moussa

**Affiliations:** ^1^ Department of Nutritional Sciences, Texas Tech University, Lubbock, TX, USA; ^2^ Obesity Research Cluster, Texas Tech University, Lubbock, TX, USA; ^3^ Center for Biotechnology and Genomics, Texas Tech University, Lubbock, TX, USA; ^4^ Department of Physiology, University of Peradeniya, Peradeniya, Sri Lanka; ^5^ Department of Biology and Biochemistry, University of Houston, Houston, TX, USA; ^6^ Department of Molecular and Cell Biology, Baylor College of Medicine Hospital, Houston, TX, USA

**Keywords:** visceral adipose tissue, subcutaneous adipose tissue, brown adipose tissue, ER stress, obesity

## Abstract

Obesity contributes to metabolic disorders such as diabetes and cardiovascular disease. Characterization of differences between the main adipose tissue depots, white (WAT) [including subcutaneous (SAT) and visceral adipose tissue (VAT)] and brown adipose tissue (BAT) helps to identify their roles in obesity. Thus, we studied depot-specific differences in whole transcriptome and miRNA profiles of SAT, VAT and BAT from high fat diet (HFD/45% of calories from fat) fed mice using RNA sequencing and small RNA-Seq. Using quantitative real-time polymerase chain reaction, we validated depot-specific differences in endoplasmic reticulum (ER) stress related genes and miRNAs using mice fed a HFD vs. low fat diet (LFD/10% of calories from fat). According to the transcriptomic analysis, lipogenesis, adipogenesis, inflammation, endoplasmic reticulum (ER) stress and unfolded protein response (UPR) were higher in VAT compared to BAT, whereas energy expenditure, fatty acid oxidation and oxidative phosphorylation were higher in BAT than in VAT of the HFD fed mice. In contrast to BAT, ER stress marker genes were significantly upregulated in VAT of HFD fed mice than the LFD fed mice. For the first time, we report depot specific differences in ER stress related miRNAs including; downregulation of miR-125b-5p, upregulation miR-143-3p, and miR-222-3p in VAT following HFD and upregulation of miR-30c-2-3p only in BAT following a HFD in mice than the LFD mice. In conclusion, HFD differentially regulates miRNAs and genes in different adipose depots with significant induction of genes related to lipogenesis, adipogenesis, inflammation, ER stress, and UPR in WAT compared to BAT.

## INTRODUCTION

Obesity is one of the most prevalent non-communicable disease that afflicts more than one-third of adults in the US [1]. It is associated with comorbid diseases including type 2 diabetes, cardiovascular disorders, stroke and cancer and is characterized by dysfunction in adipose tissue depots [2, 3]. Furthermore, secretion of adipokines and cytokines in adipose tissue is dysregulated in obesity, with increased secretion of pro-inflammatory factors and decreased section of anti-inflammatory cytokines [4].

Adipose tissue in the body is divided into smaller regional adipose depots based on structural, cellular, and biological differences between them [5]. White adipose tissue (WAT) acts as the main storage tissue for lipids and plays an important role in energy homeostasis in the body. It includes subcutaneous adipose tissue (SAT) and visceral adipose tissue (VAT) [6]. The other main adipose depot is brown adipose tissue (BAT) which produces heat by a mechanism known as thermogenesis [7]. Distribution of these adipose depots is affected by age, nutrition, gender and energy homeostasis [5]. Other important regulators that could alter genes in various conditions including obesity are microRNAs [8]. They regulate adipocyte differentiation, energy homeostasis, and immune/inflammatory system in obese conditions [9, 10]. Unbiased studies on global miRNA expression profiles along with synchronized, schematic analyses to determine mRNA and miRNA pairs between adipose depots under high fat diet-induced obesity conditions are not available to our knowledge.

Increased nutrient intake, inflammation, high demand for protein synthesis, glucose deprivation in tissues due to insulin resistance, adipocyte dysfunction and reduced vascularization are responsible for development of endoplasmic reticulum (ER) stress [3, 11]. ER stress in turn leads to accumulation of unfolded proteins in the ER known as “unfolded protein response” (UPR) [11]. Hence, we focused on adipose depot-specific differences in ER stress and UPR in obesity as there are gaps in the literature in this regard.

The aim of the present study was to identify differentially expressed genes and miRNA’s contributing to depot specific functionality in BAT, SAT and VAT in obesity. Also, we expected to identify miRNAs and genes related to ER stress and UPR pathway that are differentially regulated between the adipose depots in obesity. For these purposes, integrative analysis of comprehensive miRNA and mRNA profiles were performed in C56BL/6J (B6) mice fed on a high fat diet (HFD) to investigate depot differences in obesity. Furthermore, we validated some of adipose depot-specific differences in ER stress by comparing mice fed a HFD versus low fat diet (LFD), which was part of a larger study conducted in our lab [12]. Findings from this study would help to unravel depot specific differences, specifically in modulation of ER stress by miRNAs, to develop novel interventions to reduce ER stress and improve the metabolic problems associated with obesity.

## RESULTS

### Mice characteristics and structural depot differences

For this study, C56BL/6J mice were fed a HFD for 11 weeks and various adipose depots were dissected after sacrifice. During the study, HFD mice gained more weight over the course of 11 weeks of feeding. At end of the dietary intervention, HFD mice had significantly higher weight gain than LFD as shown in Supplementary Figure 1A (*p* < 0.05). No differences were observed in food intake between the two groups (Supplementary Figure 1B). HFD mice had significantly elevated fasting blood glucose (*p* < 0.05) (Supplementary Figure 1C). Weights of individual adipose depots and total body fat were found to be significantly higher in HFD than LFD fed mice (*p* < 0.05) as shown in Supplementary Figure 1D. Furthermore, HFD fed mice had larger adipocyte size with larger lipid droplets in all three adipose depots (VAT, SAT and BAT) than LFD mice (Supplementary Figure 2).

### Tissue specific and co-expressed transcripts and microRNAs in three adipose depots of high fat fed mice

Following RNA-Seq, ~ 24 million sequences per sample (*n* = 3 per depot) were mapped to the *Mus musculus* genome (build mm 10) and the average base call quality Q score was 38. For small RNA seq, ~ 13.8 million sequence reads per sample (*n* = 3 per depot) were mapped. Transcripts with reads per kilo base of exon model per million reads (RPKM) greater than or equal to 0.3 were considered for analysis [13]. Our biological replicates clustered together as expected (Supplementary Figure 3). BAT clearly exhibited a different transcript-miRNA expression pattern than the other two WATs (SAT and VAT). SAT and VAT demonstrated closer relationship in both hierarchical clustering of 14,213 expressed transcripts and 246 differentially expressed miRNAs respectively. Majority of the transcripts (11,865) were co-expressed in all three adipose depots of HFD fed mice. Of those, 2,472 transcripts had comparable expression levels among three adipose depots of HFD fed mice (ANOVA *p* > 0.05) and these were primarily involved in housekeeping functions as shown in Table 1.

**Table 1:** Gene ontology of co-expressed transcripts (*n* = 2,472) in VAT, SAT and BAT of HFD fed mice

	Expected number of genes	Number of genes expressed	Fold enrichment	*p* value
**Panther GO-Slim biological process**				
RNA metabolic process	226.63	326	1.44	5.62E-09
DNA-dependent transcription	184.14	228	1.39	1.90E-05
**Panther GO-Slim molecular function**				
Transferase activity	155.65	223	1.43	1.22E-05
Nucleic acid binding transcription factor activity	155.46	220	1.42	3.68E-05
DNA binding	178.46	245	1.37	7.37E-05
**Panther GO-Slim cellular component**				
Mitochondrion	10.67	23	2.16	3.20E-02
Intracellular	251.59	312	1.19	1.51E-02
**Panther GO-Slim protein class**				
Mitochondrial carrier protein	5.58	16	2.87	4.67E-02
Transferase	124.13	172	1.39	3.19E-03
Transcription factor	149.09	205	1.23	4.6-E-02

**Figure 1:**
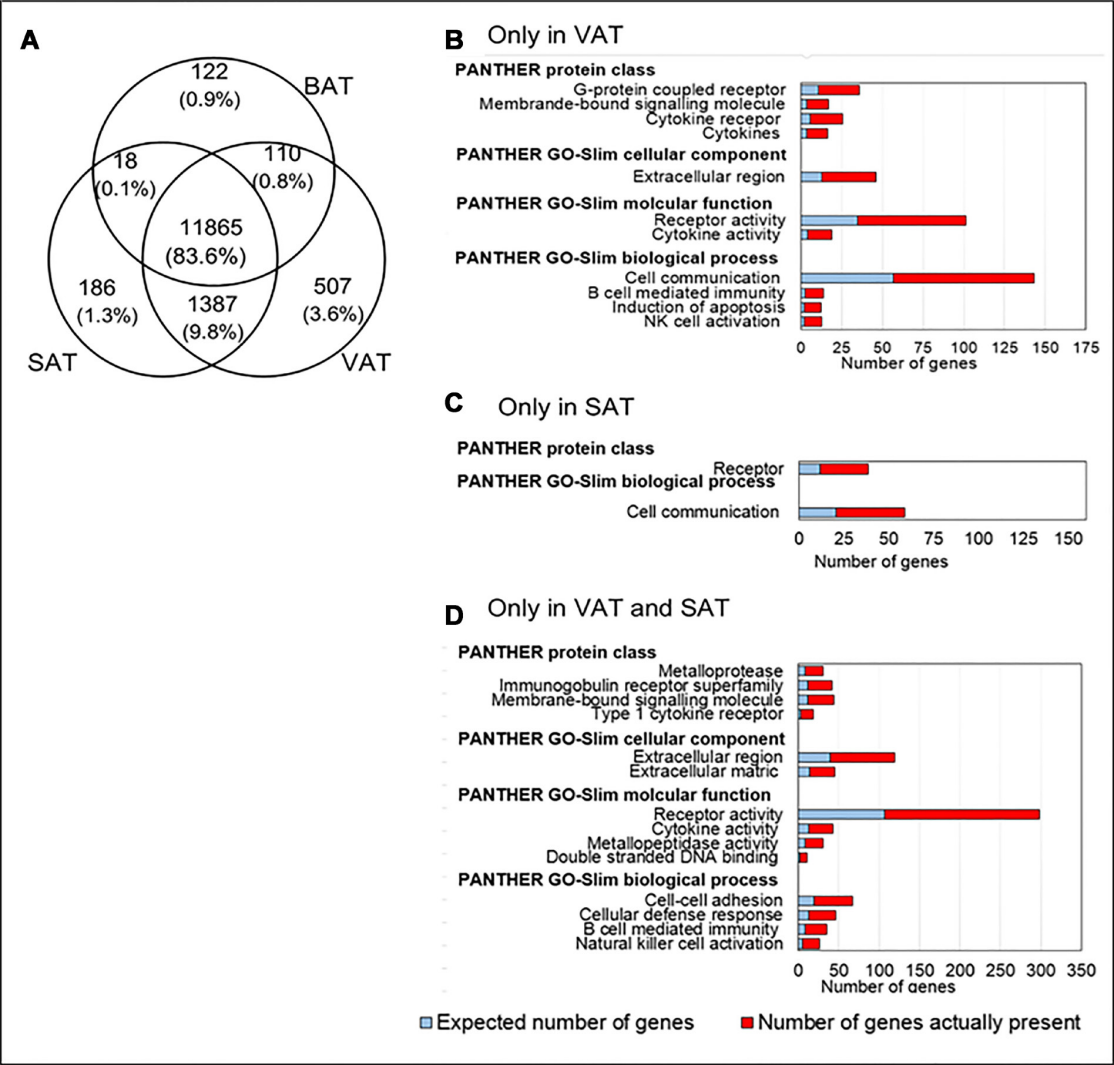
Analysis of co-expressed and tissue-specific transcripts in HFD fed mice (**A**) Venn diagram showing transcripts expressed with RPKM of ≥ 0.3 in BAT, SAT and VAT. Each depot is represented by a circle in the Venn diagram. The numbers in the various regions of the three overlapping circles indicate either unique or co-expressed transcripts among pairs of depots and among all three depots. The top significant and over represented Gene Ontology categories which include molecular functions, cellular components, biological processes, and protein classes for tissue specific and co-expressed transcripts are shown (**B**) specific to VAT, (**C**) specific to SAT, and (**D**) co-expressed in VAT and SAT are shown. The expected number of genes is based on the reference list.

We observed 13,869, 13,456, and 12,115 transcripts expressed in VAT, SAT and BAT, respectively, which is consistent with 11,000-13,000 transcripts usually expressed in human and mouse tissues [13]. In our analysis, 507, 122, and 186 transcripts were exclusively expressed in VAT, BAT and SAT, respectively (Figure 1A). According to gene ontology (GO) analysis, VAT-specific transcripts were related to inflammatory functions such as cytokine activity, B cell mediated immunity, natural killer cell activities, cell communication and induction of apoptosis processes (Figure 1B). Transcripts specific to SAT were related to cell communication and receptor proteins (Figure 1C), while no specific function was observed by GO analysis for transcripts specific to BAT. The co-expressed transcripts in SAT and VAT were related to cell-cell adhesion, cellular defense response, B cell mediated immunity, and natural killer cell activation, and were suggestive of inflammatory response (Figure 1D).

**Figure 2:**
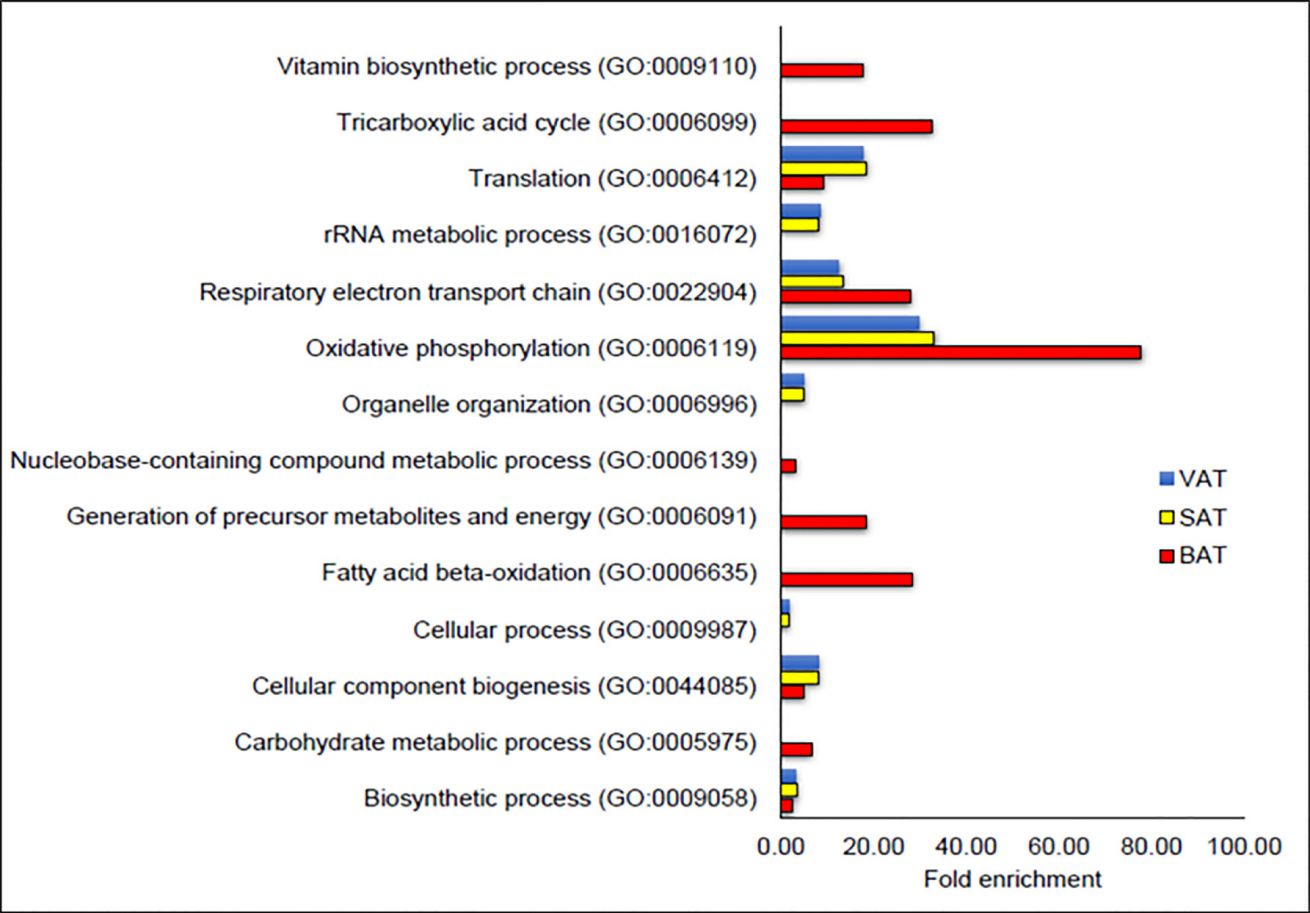
Gene Ontology (GO)-Biological functions of highly expressed genes in adipose depots in HFD fed mice Fold enrichment scores for the significant, over represented biological processes of highly expressed transcripts with RPKM of 500 or more in BAT, SAT and VAT in high fat diet fed mice based on GO analysis. (High levels of expression (> 500 RPKM) was observed for 202, 252 and 248 transcripts in BAT, SAT and VAT, respectively).

We performed gene ontology analysis to identify the biological functions of the highly expressed transcripts in each depot (Figure 2).

### Differentially expressed transcripts and miRNAs in pairwise comparisons in high fat fed mice

C-C motif chemokine ligand 8 *(Ccl8),* serpin family A member 3 *(Serpina3n),* WAP four-disulfide core domain 21 *(Wfdc21),* secreted frizzled-related protein 5 *(Sfrp5),* and matrix metallopeptidase 3 *(Mmp3)* transcripts were among the top differentially expressed transcripts, with higher expression in WAT (both VAT and SAT) than in BAT. Asparaginase *(Aspg),* cell death-inducing DFFA-like effector A *(Cidea),* uncoupling protein 1 *(Ucp1)*, and family with sequence similarity 151 member A *(Fam151a)* had higher expression in BAT than in WAT (SAT and VAT) (Supplementary Tables 1 and 2). In VAT/SAT comparison, only 3 transcripts, enoyl-CoA hydratase domain containing 2 (*Echdc2),* Tumor necrosis factor (TNF) alpha induced protein 8 like 1 *(Tnfaip8l1),* and Src homology 2 domain containing F *(Shf)* had significantly higher expression in SAT than in VAT (Supplementary Table 3).

**Table 2:** Top canonical pathways related to adipose tissues in the VAT/BAT comparison following a HFD

Activated canonical pathways in VAT relative to BAT	-log (*p*-value)	Ratio	*z*-score	Differential/total genes
Integrin Signaling	15.300	0.479	8.342	105/219
NRF2-mediated Oxidative Stress Response	14.300	0.487	5.571	94/193
B Cell Receptor Signaling	13.700	0.486	7.117	90/185
IL8 Signaling	12.600	0.467	8.786	92/197
PI3K/AKT Signaling	12.500	0.532	3.357	66/124
Fcγ Receptor-mediated Phagocytosis in Macrophages and Monocytes	12.400	0.581	7.076	54/93
PI3K Signaling in B Lymphocytes	12.200	0.523	6.786	67/128
mTOR Signaling	10.900	0.447	5.422	89/199
NGF Signaling	10.500	0.513	7.353	60/117
PDGF Signaling	9.930	0.544	6.143	49/90
**Inhibited canonical pathways in VAT relative to BAT**	**-log (*p*-value)**	**Ratio**	***z*-score**	**No of genes**
PTEN Signaling	8.600	0.479	−4.160	57/119
RhoGDI Signaling	8.580	0.434	−5.500	75/173
PPARα/RXRα Activation	6.420	0.399	−2.655	71/178
PPAR Signaling (PPARα)	2.950	0.376	−4.226	35/93
Role of p14/p19ARF in Tumor Suppression	2.750	0.442	−3.300	19/43

–log *p* value > 1.3 is equivalent to *p* value < 0.05 and is significant. Ratio is number of differentially expressed/total number of genes in a canonical pathway. *Z* score ≤ −2 is inhibited and ≥ 2 is activated. Abbreviations: AKT (protein kinase B), ARF (alternative reading frame), IL8 (Interleukin-8), PI3K (phosphoinositide 3-kinase), mTOR (mechanistic target of rapamycin), NGF (nerve growth factor), NRF2 (nuclear factor-erythroid 2-related factor), PPAR (peroxisome proliferator activated receptor), PDGF (platelet-derived growth factor), PTEN (phosphatase and tensin homolog), RhoGDI (Rho GDP-dissociation inhibitor), and RXR (Retinoid X receptor).

We also identified 1,251 miRNAs, out of which 246 were differentially expressed with a fold change of more than 1.25 and *p* value < 0.05 in the pairwise comparisons of the 3 adipose depots of high fat fed mice indicating that miRNAs play different roles in different adipose depots with HFD. The top 10 differentially expressed miRNAs included miR-193b-5p, miR-365-1-5p, miR-129-5p, miR-122-5p, miR-6240 and miR-5130 which had lower expression in both SAT and VAT (WAT) than in BAT. MiR-335-3p, miR-223-3p, miR-340-5p, miR-298-5p and miR-224-5p had higher expression in SAT and VAT than in BAT (Supplementary Table 4).

IPA^®^ analysis was used to identify significant canonical pathways altered by high fat diet. Z score of 2 or more indicates that the canonical pathway is activated based on the differentially expressed transcripts in the dataset. Of the 312 canonical pathways identified in the VAT/BAT comparison, 148 canonical pathways were predicted to have significantly higher activity in VAT than in BAT, while 6 were predicted to have significantly higher activity in BAT than VAT (top pathways are listed in Table 2). In the VAT/BAT comparison, cytokine related pathways including TNF related weak inducer of apoptosis (TWEAK) signaling, integrin signaling, interferon signaling and TNF receptor (TNFR1 and 2) signaling pathways had higher percentage of enrichment by differentially expressed transcripts and were predicted to be activated in VAT than BAT (*p* < 0.05, Z score ≥ 2). In contrast, phosphatase and tensin (PTEN) signaling and peroxisome proliferator activated receptor (PPARΑ)/retinoid X receptor (RXR-Α) pathways were predicted to have a higher activity in BAT than in VAT (z score ≤ -2) (Table 2).

Similar to the previous assessment, 312 pathways were significant in the IPA^®^ based analysis for SAT/BAT comparison. Of those, 147 were predicted to be significantly activated, while 6 canonical pathways were predicted to be significantly inhibited in SAT than in BAT (top pathways are listed in Supplementary Table 5). Overall pathway activation or inhibition was not predicted by IPA^®^ for VAT/SAT comparison, since the number of differentially expressed transcripts was less and only few transcripts enriched the canonical pathways (Supplementary Table 6).

We identified the top molecular and cellular functions of the differentially expressed miRNAs in BAT/VAT comparison (Table 3). These include cell proliferation, cell death and migration and are in line with the canonical pathways enriched by the differentially expressed mRNA in the BAT/VAT comparison. Similarly, differentially expressed miRNAs in VAT/SAT comparison echoed the biological functions of differentially expressed transcripts in the same comparison (Supplementary Table 7).

**Table 3:** Top disease and functional categories of differentially expressed miRNAs in the VAT/BAT comparison of HFD fed mice

Diseases or functions annotation	*p*-Value	Activation *z*-score	Number of molecules
**Cellular growth and proliferation**			
Proliferation of cells	1.04E-03	−0.736	38
Cell proliferation of tumor cell lines	2.77E-11	−0.549	34
**Cell death and survival**			
Necrosis	1.85E-02	1.273	25
Cell death of tumor cell lines	2.95E-02	1.421	16
**Cancer, organismal injury and abnormalities**			
Advanced malignant tumor	1.72E-06	0.447	19
Neoplasia of cells	4.53E-07	1.191	43
Metastasis	1.60E-06	0.447	18
**Cellular movement**			
Cell movement of tumor cell lines	4.37E-06	0.776	18
Migration of tumor cell lines	1.38E-06	1.466	17
Invasion of tumor cell lines	4.57E-07	0.157	17

*P* value < 0.05 is significant.

Furthermore, according to functional analysis using IPA^®^, VAT/BAT and SAT/BAT comparisons had almost similar canonical pathway activities, upstream regulators, and biological functions. Hence, we focused on comparing VAT/BAT of HFD fed mice for further functional analysis, since VAT/BAT comparison could represent WAT/BAT comparison.

### Adipose depot specific differences in high fat diet induced ER stress

**Figure 3:**
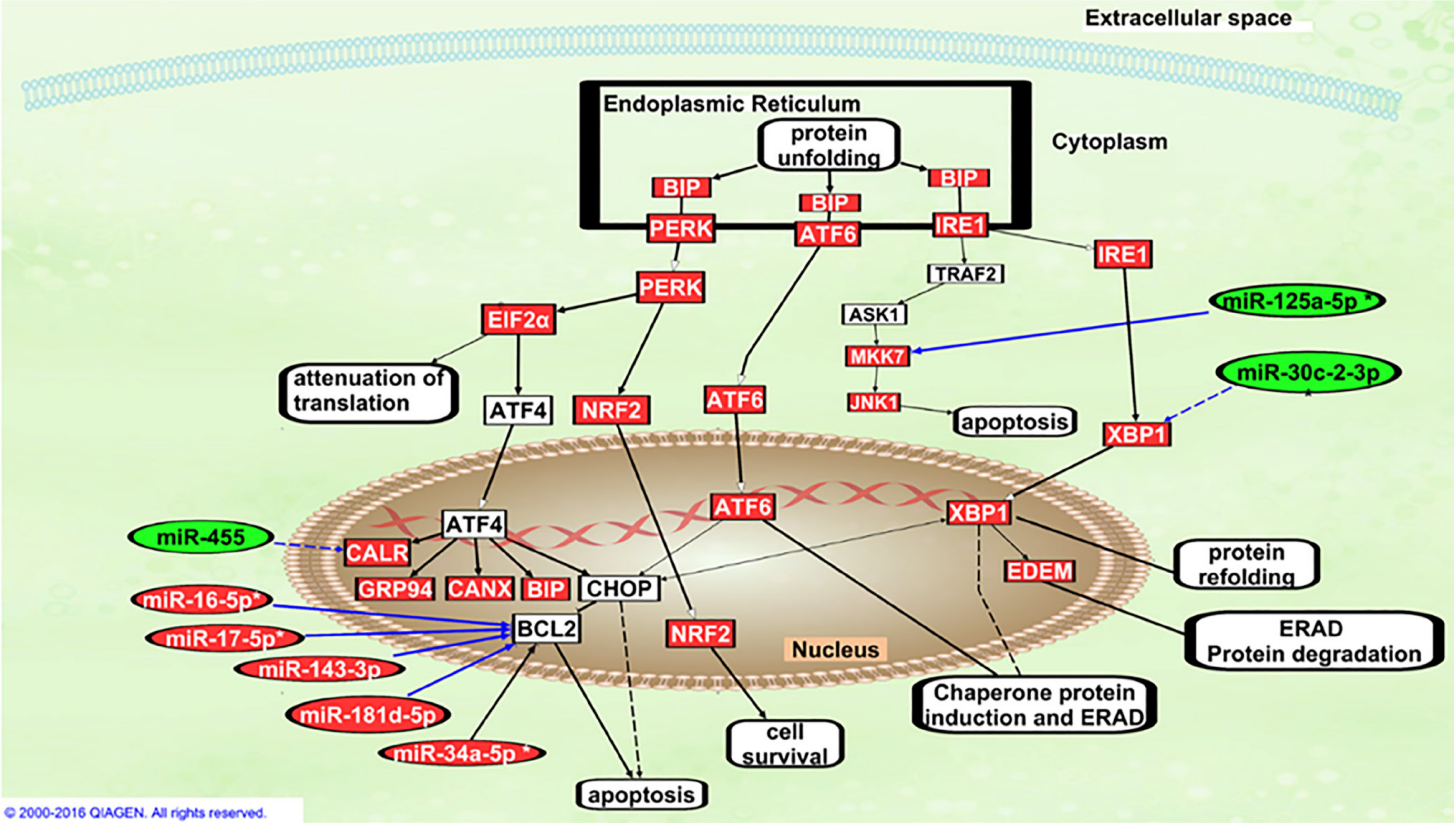
Differentially expressed genes and miRNAs related to ER Stress and Unfolded Protein Response pathway in the VAT/BAT comparison of HFD fed mice using Ingenuity Pathway Analysis (IPA^®^) Genes and miRNAs with higher expression in VAT than in BAT are indicated in red, while those with lower expression in VAT than in BAT are in green. Complete lines indicate direct relationships and dashed lines indicate indirect relationships.

Following RNA-Seq analysis of adipose depots from HFD fed mice, we narrowed our focus on ER stress/UPR pathway as it is a major complication caused by numerous factors in obesity [11]. ER stress pathway was among the top 20 significant canonical pathways identified by IPA^®^ in the VAT/BAT comparison of HFD fed mice. Even though the overall activity was not predicted for UPR or ER stress pathways by IPA^®^, sixteen (76.2% of total genes in ER stress pathway) transcripts related to ER stress pathway had higher expression in VAT than in BAT of HFD fed mice (Figure 3). Similarly, SAT also had higher expression of 11 ER stress related transcripts than BAT in HFD fed mice. Furthermore, X-box binding protein 1 *(Xbp1),* an ER stress marker had significantly higher expression in both SAT and VAT (*p* < 0.05) than in BAT. Interestingly, we were able to identify 8 differentially expressed miRNAs related to UPR including miR-30c-2-3p, miR-455, miR-125a-5p, miR-17-5p, miR-143-3p, miR-16-5p, miR-181d-5p and miR-34a-5p in VAT/BAT based on literature and IPA^®^ (Figure 3). These findings suggest higher ER stress and UPR activity in WAT (SAT and VAT) than in BAT in high fat fed mice.

### Validation of RNA-Seq and small RNA-Seq results with quantitative polymerase chain reaction (qPCR) in adipose tissue of HFD fed mice

**Figure 4:**
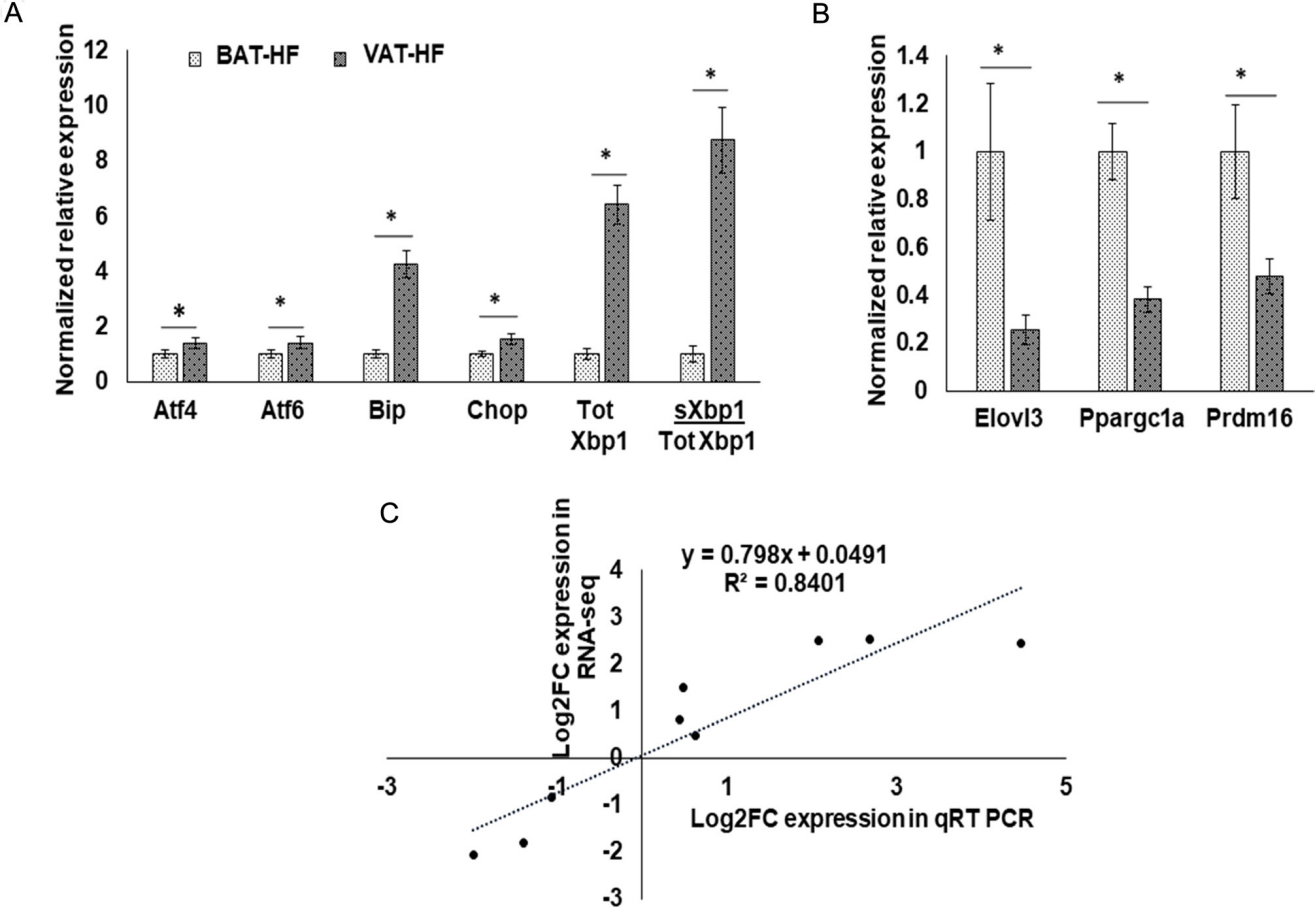
Validation of RNA-Seq data using RT-qPCR for VAT/BAT of HFD fed mice Data are expressed as mean ± standard error of mean (SEM). (**A**) Gene expression of ER stress markers, *Atf4, Atf6, Bip, Chop, Xbp1(Tot),* and *Xbp1(s)/Xbp1(Tot)*. ^*^ indicate ANOVA *p* < 0.05, *n* = 3 per group. (**B**) Typical BAT markers such as *Elovl3, Ppargc1a*, and *Prdm16*. ^*^indicate ANOVA *p* < 0.05, *n* = 3 to 4 per group. (**C**) Scatter plot for log2 fold changes (Log2FC) in the BAT/VAT comparison obtained from RNA-Seq and RT-qPCR. (Pearson correlation coefficient (*r*) = 0.917, *p* value = 0.001).

We validated our RNA-Seq findings with qPCR. We observed significantly higher expression of activating transcription factor 4 *(Atf4),* activating transcription factor 6 (*Atf6)*, heat shock protein family A (Hsp70) member 5 *(Bip),* C/EBP-homologous protein *(Chop),* total *Xbp1,* and soluble *Xbp1/*total *Xbp1* ratio in VAT than BAT of HFD fed mice with qPCR (Figure 4A). Even though *Atf4* and *Chop*, had 1.7 and 1.4 fold change, respectively with qPCR, these were not among the differentially expressed transcripts in the RNA-Seq analysis (*p >* 0.05). The BAT markers, ELOVL fatty acid elongase 3 *(Elovl3)*, peroxisome proliferator activated receptor gamma co-activator-1a *(Ppargc1a)* and PR domain 16 *(Prdm16),* showed significantly higher expression in BAT compared to WAT (*p* < 0.05) as shown in Figure 4B. Thus, our qPCR findings were consistent with our RNA-Seq data as shown by two-tailed Pearson correlation (*r* = 0.917, *p* value = 0.001) (Figure 4C).

We performed qPCR for selected miRNAs to validate our small RNA sequencing for VAT/BAT comparison. Same direction of fold change was observed for seven miRNAs including miR-455-3p, miR-30c-2-3p, miR-222-3p, miR-99b-5p, miR-199a-3p, miR-143-3p, and let-7a-5p. Fold changes for miR-708-5p, miR-30a-5p and miR-221-3p with qPCR were in the opposite direction than that of small RNA sequencing. The correlation was not statistically significant probably because we tested only a few miRNAs of interest (Pearson correlation coefficient = 0.52, *p* value = 0.23).

**Figure 5:**
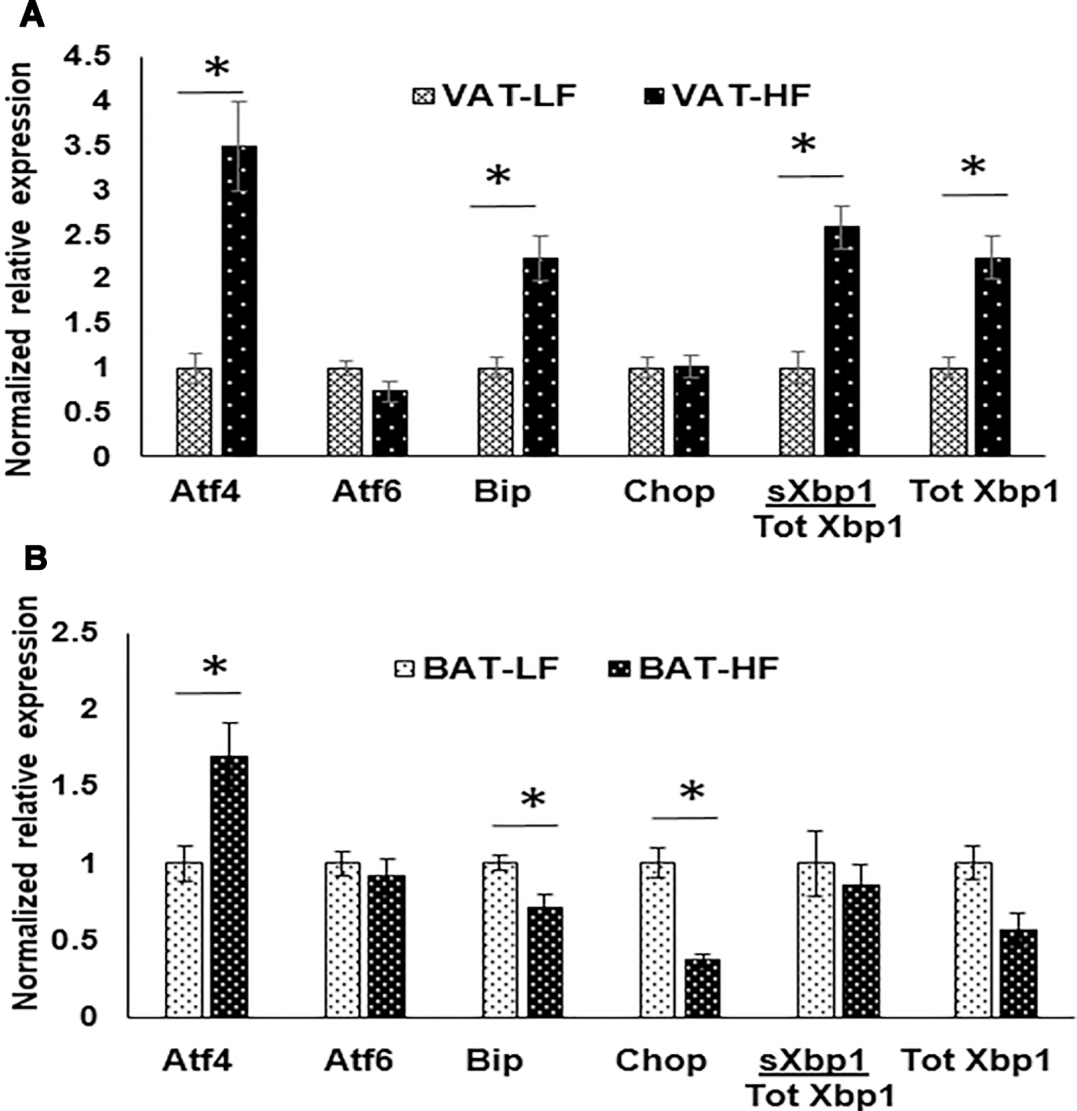
Normalized expression of ER stress markers in VAT and BAT with HFD vs LFD mice comparison using RT-qPCR (**A**) in VAT (**B**) in BAT ^*^indicates *p* value < 0.05, *n* = 3 per group. 18S was used as the house keeping gene for normalization. BAT- HF (BAT from mice fed on a HFD), BAT-LF (BAT from mice fed on a LFD), VAT-HF (VAT from mice fed on a HFD), VAT-LF (VAT from mice fed on a LFD).

To further understand the adipose depot differences in HFD-induced ER stress, we studied differences in ER stress markers between HFD and low fat (LF) diet fed mice using both BAT and VAT tissues. With our qPCR analysis, we noted depot specific differences in expression of ER stress related genes (Figure 5). *Atf4, Bip, Total Xbp1*, and *sXbp1/Total Xbp1* ratio were all upregulated significantly (*p* values < 0.05) in VAT-HF than in VAT-LF, while *Chop* and *Atf6* did not show any significant change but were trending with *p* values of 0.052 and 0.938, respectively (Figure 5A). However, a different gene expression pattern was observed in BAT-HF vs BAT-LF. Only *Atf4* was significantly upregulated (*p* < 0.05) while, *Bip* and *Chop* were significantly down regulated in BAT-HF than in BAT-LF (*p* < 0.05) as shown in Figure 5B.

**Figure 6:**
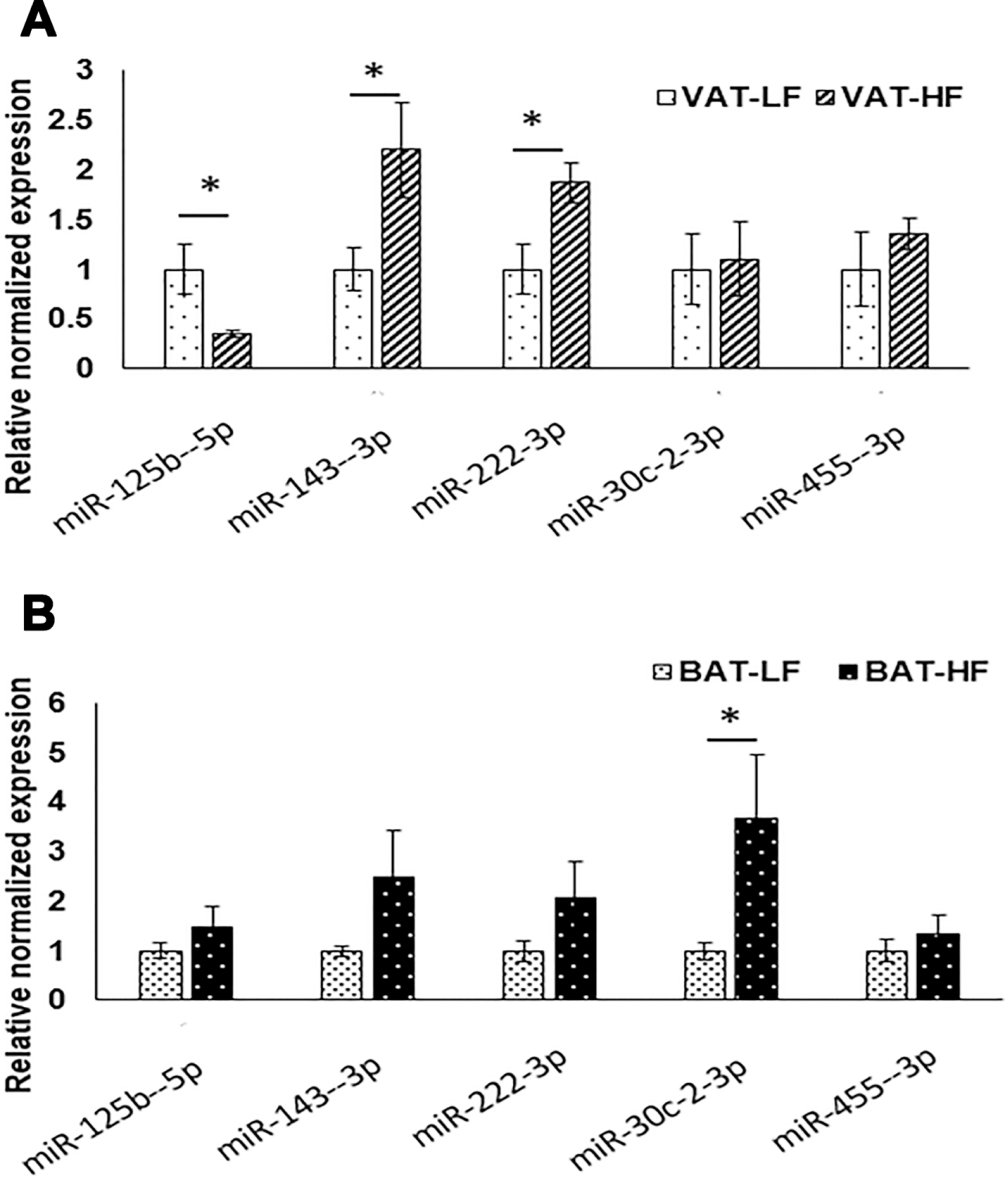
ER stress related miRNA expression following LFD and HFD in mice using RT-qPCR (**A**) in VAT and (**B**) in BAT, ^*^indicates *p* value < 0.05, *n* = 4 to 6 per group. miR-191-5p was used for normalization.

We also performed qPCR for selected miRNAs that were related to ER stress (miR-125b-5p, miR-143-3p and miR-222-3p, miR-30c-2-3p, and miR-455-3p), in BAT and VAT of LFD and HFD fed mice (Figure 6). MiR-143-3p and miR-222-3p were significantly upregulated (*p* < 0.05), while miR-125b-5p was significantly down regulated (*p* < 0.05) in VAT-HF than VAT-LF (Figure 6A). Interestingly, miR-30c-2-3p was differentially expressed only in BAT and was significantly upregulated (*p* < 0.05) in BAT-HF than BAT-LF. Mir-143-3p and miR-222-3p showed an increasing trend due to HFD in BAT similar to VAT, but were not statistically significant (*p* value = 0.077 and 0.118) (Figure 6B). Finally, we combined our IPA^®^ analyses and previous literature to identify miRNA-mRNA associations related to ER stress and UPR pathway. Furthermore, using the molecular activity prediction feature, we predicted other differentially expressed genes in the UPR pathway (Supplementary Figures 4 and 5).

## DISCUSSION

**Figure 7:**
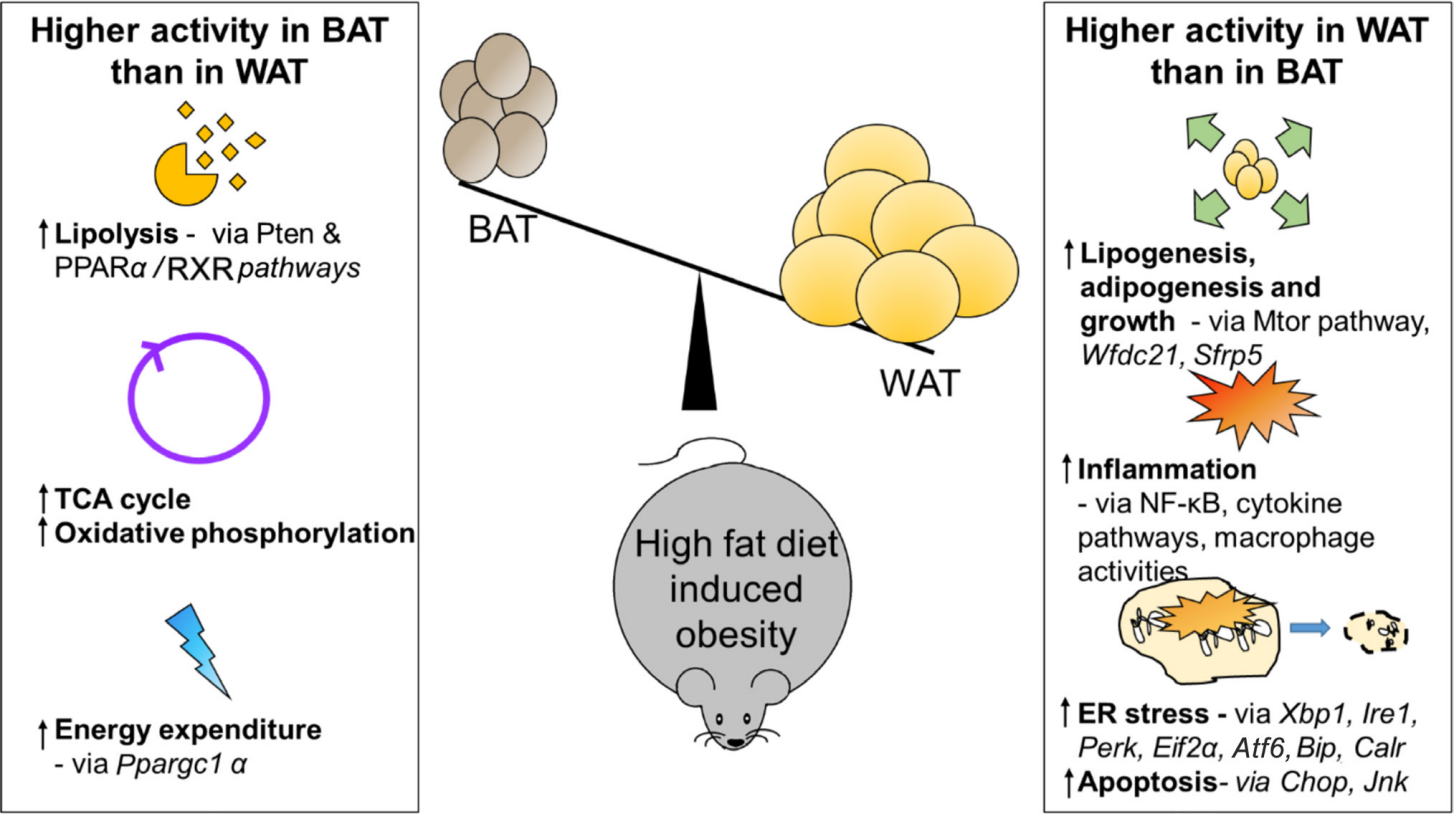
Comparison of white and brown adipose tissue in high fat diet induced obesity in mice-summary of the findings As a summation of the results from combined analysis of RNA-Seq and miRNA profiling, the differential expression or activity of pathways and genes in brown (BAT) and white (WAT) adipose tissue in high fat fed obese mice are shown here. Some pathways and genes are quoted as examples.

Adipose depot specific differences and their relative contribution to HFD induced obesity and its associated metabolic derangements are not clear. Our primary objective was to identify obesity related adipose depot differences. For this purpose, we compared VAT, SAT and BAT of high fat diet fed obese male mice. Extensive literature from our lab and others show that male mice are more prone to diet induced obesity and its complications, while female mice are protected from obesity [14]. To our knowledge, our study is the first to observe adipose depot-specific differences in global mRNA and miRNA expression in BAT, SAT and VAT in HFD-induced obesity. Novel findings from our report include transcriptomic comparisons of WAT (SAT and VAT) vs BAT from HFD fed obese mice (summarized in Figure 7). Moreover, we identified novel adipose depot-specific differences in HFD-induced ER stress-related genes and miRNAs.

Our findings support the notion that WAT functions mainly as an energy storage [5]. *Sfrp5* and *Wfdc21* were among the top upregulated transcripts in VAT than in BAT of HFD fed mice in our study. *Sfrp5* stimulates adipocyte growth during obesity by inhibition of Wnt signaling [15]. *Sfrp5* expression is significantly downregulated when the mice are fed on an 30% energy restricted diet [16]. *Wfdc21* is highly expressed in WAT than in BAT and is involved in remodeling of the extracellular milieu in adipogenesis [17, 18]. Furthermore, mTOR pathway which is involved in adipogenesis and lipogenesis [19] was predicted to have a higher activity in WAT (SAT and VAT) than in BAT. This corroborates with lower PTEN activity in WAT than in BAT, since PTEN pathway inactivates mTOR signaling [20]. Additionally, miR-129-5p which is down-regulated during 3T3-L1 preadipocyte proliferation [21], had lower expression in VAT than BAT of HFD fed mice in our study. Thus, it is plausible that adipogenesis is higher in WAT (SAT and VAT) than in BAT of HFD mice.

Based on our observations, both types of WATs have more inflammation than BAT in obesity. The top differentially expressed transcripts and pathways observed in WAT were pro-inflammatory. Inflammation and obesity associated miR-335 and miR-223 [22, 23] had higher expression in VAT than in BAT in HFD fed mice in our study. Furthermore, we observed that the VAT-specific transcripts were related to inflammation suggesting that VAT has higher inflammation with HFD as previously shown [24–26]. Additionally, higher cellular stress was observed in VAT than in BAT in HFD fed mice. Apoptosis related transcripts including *Jnk,* and *Chop* had higher expression in VAT than BAT suggesting UPR is being directed towards apoptosis in VAT in HFD-induced obesity. Hence, our findings provide a rationale for association between VAT and higher metabolic risk [27], highlighting the importance of reducing VAT in obesity treatment.

In our study, highly expressed transcripts (RPKM of 500 or more) in BAT of HFD fed mice were related to mitochondrial metabolic activities including tricarboxylic acid cycle, oxidative phosphorylation, respiratory electron transport chain and fatty acid oxidation which are the main biological functions of BAT. Furthermore, miR-129-5p which is involved in browning process [28] had higher expression in BAT than VAT of the HFD mice in our study. Additionally, lipolysis related *Aspg* [29], and fatty acid oxidation-related PPARA and PTEN canonical pathways [30, 31] were elevated in BAT more than VAT of HFD fed mice indicating BAT to have a higher ability to “burn the FAT” in obesity. Therefore, further enhancement of the existing BAT activity may be a potential treatment option for obesity.

We report for the first time, adipose depot differences in expression of ER stress related miRNAs. MiR-30c-2-3p which targets *Xbp1* [32] was specifically upregulated in BAT due to HFD induced obesity. Thus, upregulation of miR-30c-2-3p may reduce cell survival in BAT in HFD fed mice and this may be linked to brown adipocyte dysfunction that is known to occur with obesity [33]. We observed depot-specific downregulation of miR-125-5p only in VAT of HFD than LFD mice. MiR-125b-5p is degraded by activation of IRE1α in ER stress [32]. Thus, activation of IRE1α by HFD-induced ER stress may lead to reduced expression of miR-125b-5p, which may cause increased levels of *Casp2* and *Mkk7,* and promote apoptosis in VAT of HFD fed mice (Supplementary Figure 4). Also, miR-143-3p and miR-222-3p were upregulated in VAT of HFD than LFD mice in our study. Significant upregulation of miR-143-3p in VAT may increase cell death via repression of *BCL2* [34]. Additionally, upregulated miR-222 in VAT-HF than BAT-HF in our study is known to increase ER stress-mediated apoptosis in liver cells via inhibition of *CDKN1B* (p27Kip1) [35]. Thus, upregulation of miR-222-3p may cause *Cdkn1b*: miR-222-3p mediated apoptosis in VAT in obesity. Another miRNA, miR-455: calreticulin association was identified previously in murine cardiomyocytes [32] However, we did not observe any significant changes in miR-455-3p in adipose tissue. This may be because some of the miRNA: mRNA associations are tissue specific. We did not measure ER stress and apoptosis related proteins since that is not within the scope of the current study. However, depot specific differences in expression of ER stress related miR-30c-2-3p, miR-125b-5p, miR-143-3p and miR-222-3p in HFD induced obesity are novel findings, but these are yet to be confirmed in clonal adipocytes.

While the current study provides comprehensive information on three different adipose tissue depots in a unique manner to identify depot-specific differences between different adipose depots following a HFD, there are few limitations. We only used adipose depots from HFD fed mice for RNA-seq and Small RNA-sequencing and did not have a LFD group for this analysis. This is because our aim was to study global mRNA and miRNA expression of different adipose tissue depots in dietary obesity. Nevertheless, a low-fat fed control group to compare diet induced effects at whole transcriptome and miRNA profile level would have provided additional insights. However, we used LFD mice as a control group to study depot differences in ER stress related gene and miRNA expression.

## CONCLUSIONS

In summary, we observed depot specific differences in global gene and miRNA expression in HFD-induced obesity. SAT and VAT were more closely related to each other than BAT in obesity. Based on our transcriptomic analysis of adipose depots of HFD fed mice, WAT (SAT and VAT) has higher adipogenesis, lipogenesis, inflammation, cellular stress and ER stress than in BAT. BAT has higher expression of transcripts related to oxidative metabolism and lipolysis than WAT with HFD-induced obesity. For the first time, we report depot specific differences in ER stress related miRNAs including; downregulation of miR-125b-5p and upregulation of miR-143-3p, and miR-222-3p in VAT following HFD and upregulation of miR-30c-2-3p only in BAT following a HFD in mice.

## MATERIALS AND METHODS

### Experimental animals and tissue collection

Animal studies used for the present study have been previously described [12]. Briefly, male C57BL/6J mice aged 5–6 weeks were acclimated for one week. These obesity prone mice were fed on a HFD (45%, 20% and 35% of energy from fat, protein and carbohydrate, respectively, *n* = 10) or a LFD (10%, 20% and 70% of energy from fat, protein and carbohydrates, respectively, *n* = 9) for 11 weeks (Detailed diet information is in Supplementary Table 8) [12]. At the end of 11 weeks, mice were fasted, and sacrificed using CO_2_ inhalation method. Epididymal fat (VAT), inguinal fat (SAT) and interscapular brown fat (BAT) were harvested, snap frozen immediately in liquid nitrogen, and stored at −80° C for further analyses. The protocols were approved by the Institutional Animal Care and Use Committee of the University of Tennessee, Knoxville. The outline of the study is shown in Supplementary Figure 6.

### RNA extraction

RNA was extracted from frozen adipose tissue samples (SAT, VAT and BAT) according to manufacturer’s protocol with RNeasy lipid tissue kit (QIAGEN, Redwood City, CA). RNA was quantified using a spectrophotometer (Nanodrop, Thermo Scientific, Waltham, MA, USA) by measuring absorbance (nm) at 260/280. RNA quality of SAT, VAT and BAT of HFD mice were evaluated using Agilent 2200 Tape station (Agilent Technologies, Santa Clara, CA). Samples with RNA integrity numbers (RIN) between 7.1 to 7.6 were used for RNA sequencing.

### Library preparation and RNA sequencing

We performed RNA-Seq and Small RNA-Seq using RNA from SAT, VAT and BAT of HFD fed mice (*n* = 3 per adipose depot type) only. RNA sequencing libraries were prepared using Illumina TruSeq RNA sample preparation kit v2-Low sample protocol (Illumina, Inc., San Diego, CA). Briefly, mRNAs were purified from 2–4 μg of RNA using oligo-dT attached magnetic beads. Then, mRNAs were fragmented and copied into first strand cDNA using reverse transcriptase and random primers. Synthesized double stranded cDNA were recovered using Agencourt, AMPure XP beads (Beckman Coulter, USA) and cDNA fragments were end repaired. This was followed by multiplexing using 3′ adenylation and adapter (with indexes) ligation, purification of adaptor-ligated fragments and enrichment using PCR. Final cDNA libraries were validated and insert size identified using Agilent 2200 Tape station. They were quantified using Qubit 2.0 fluorimeter (Invitrogen, Life Technologies). Multiplexed cDNA libraries were diluted, denatured and loaded on to a HiSeq Rapid flow cell. Paired end sequencing with 108 bp read length was performed on Illumina HiSeq 2500 (Illumina, Inc., San Diego, CA) at the Center for Biotechnology and Genomics Core Facility at Texas Tech University.

Small RNA libraries were prepared using the Illumina small RNA protocol (Illumina, Inc., San Diego, CA) and small RNA sequencing was performed using the Illumina Genome Analyzer NextSeq 500 (Illumina, Inc., San Diego, CA) at the Department of Molecular and Cell Biology, Baylor College of Medicine, Houston.

### Mapping and assembly

Reads from VAT, SAT and BAT of HFD mice were mapped to the NCBI mouse genome build 10 (*Mus musculus*-GRCm38.p4). FastQC High Throughput Sequence QC report (Version 0.11.2, (http://www.bioinformatics.babraham.ac.uk/projects/fastqc/) was used to check the quality of raw reads of each sample [36]. Assembly and mapping following RNA sequencing was performed using QSeq^®^. Version 12. (DNASTAR. Madison, WI). The Gunaratne Next Generation pipeline was utilized to determine miRNA expression profiles [37]. Briefly, RNA-Seq reads were mapped using TopHat2 [38] onto the mouse genome build UCSC mm10, and gene expression was quantified using Cufflinks2. Differential reads were determined applying a parametric *t*-test, with significance achieved at *p* < 0.05 and fold change exceeding 1.25× in either direction. Small RNA-Seq reads were mapped against the miRbase [39] reference onto miRNA precursors, and reads mapping on mature miRNAs were selected. MiRNA coverage was assessed with reads mapping to multiple locations being accounted for proportionally for each mapping. Finally, we applied the SigTerms pipeline [40], looking for enriched miRNA /mRNA interactions, with significance achieved at FDR-adjusted *p*-value < 0.25.

For miRNAs, counts of each unique read were normalized to total usable reads and 40 counts were added. Expression value for each transcript and miRNA was stated as RPKM.

### Data availability

Data for RNA-Seq for each of the three biological replicates per adipose depot of HFD mice were deposited at NCBI Sequence Read Archive (SRA) submission under Bioproject PRJNA341297. Individual accession numbers for the three BAT samples are SAMN05717642, SAMN05717643 and SAMN05717644; accession numbers for the SAT are SAMN05717645, SAMN05717646 and SAMN05717647; accession numbers for the VAT are SAMN05717648, SAMN05717649 and SAMN05717650 (http://www.ncbi.nlm.nih.gov/bioproject/). RNA-Seq data for miRNA profiling has been submitted to the Gene Expression Omnibus (GEO) data repository (http://www.ncbi.nlm.nih.gov/projects/geo/) under accession number GSE85101.

### Functional analysis of data following RNA-Seq and Small RNA-Seq

### Tissue specific transcripts in adipose depots of HFD fed mice

A value of 0.3 RPKM was used to filter transcripts since it is considered the threshold for detectable expression above background [13]. Hierarchical clustering was performed using TIGR Multiple Experiment Viewer (MeV) v4.8.1 for expressed transcripts and for differentially expressed miRNAs [41]. Common and depot specific genes were identified using Venny- which is web application for comparison and visualization of biological lists using expressed transcripts with a RPKM of 0.3 or more in each depot [42]. GO analysis, including molecular functions, cellular components, biological processes, and protein classes, was studied in co-expressed and depot-specific transcripts using www.geneontology.org [43]. The genome of *Mus musculus* was used as background. In this program, the expected number of genes is based on the reference list. Fold enrichment of the genes observed is the ratio of transcripts in the uploaded list over the expected. A ratio greater than 1 implies that the category is overrepresented in the experimental data. *P*-value is the probability that the number of genes observed in these categories occurred by chance calculated with the binomial distribution formula. *P* < 0.05 is considered significant, with Bonferroni correction. For further classification of transcripts expression in the 3 adipose depots, we categorized the results into four groups: high (≥500 RPKM), medium (1 to 500 RPKM), low (0.3 to 1 RPKM), and very low expression (<0.3 RPKM) [13, 44, 45].

### Differentially expressed genes and miRNAs in HFD fed mice

Pair wise comparisons were performed as follows for adipose depots of HFD fed mice: 1) VAT/BAT (BAT as control), 2) SAT/BAT (BAT as control) and 3) VAT/SAT (SAT as control). Differentially expressed transcripts with 2 or more fold change at 95% confidence were selected with moderated *t*-test at a false discovery rate of 5% (using the Benjamini & Hochberg FDR test). In addition, ANOVA (*F*-test) was used to compare all 3 adipose tissues. *P* < 0.05 was considered significantly different. DNA Star QSeq software was used to perform the above statistical tests. Differentially expressed miRNAs between depots were identified by Student’s *t*-test with a fold change of more than 1.25 or more.

Differentially expressed transcripts with expression greater than or equal to 1 RPKM (to avoid genes with low expression) and differentially expressed miRNAs were submitted to QIAGEN’s Ingenuity Pathway Analysis^®^ (IPA^®^, QIAGEN Redwood City, CA; www.qiagen.com/ingenuity) core analysis for VAT/BAT, SAT/BAT, and VAT/SAT comparisons of HFD fed mice (Supplementary Figure 7). Using the IPA^®^ Knowledge Base, canonical pathway analyses, upstream regulators, and regulatory pathways were generated. In the canonical pathway analyses, Fischer’s Exact test’s *P* values indicate the significance of enrichment of pathways by differentially expressed transcripts. The Z score indicates if the canonical pathway is activated or inhibited based on the differentially expressed transcripts in the dataset. Upstream regulator analysis predicts upstream regulators based on the transcript expression data using the Ingenuity^®^ Knowledge Base. A Fisher’s exact test calculates the *P*-value of overlap, which indicates the significance of enrichment of the gene expression data for genes downstream of an upstream regulator. Differentially expressed miRNAs that were experimentally validated and directly related to canonical pathways were added using the Grow function in IPA^®^.

### Quantitative real-time polymerase chain reaction

### For validation of RNA-Seq data and to study depot differences in ER stress

cDNA was made from RNA according to manufacturer’s recommendation using the iScript™ cDNA Synthesis Kit (Bio-Rad Laboratories, Inc. CA, USA) and quantitative real-time polymerase chain reaction (qPCR) was performed using the BioRad CFX-96 real time PCR detection system (Bio-Rad Laboratories, Inc. CA.USA) with SYBR-Green Real-time PCR Master Mix (Bio-Rad Laboratories, Inc. CA, USA). For validation of RNA-Seq results, we measured expression of a few BAT marker genes ELOVL fatty acid elongase *(Elovl3), Pgc1a,* and *Prdm16*, as well as ER stress markers genes including total *Xbp1(Tot)*, spliced *Xbp1(s), Bip, Atf4, Atf6,* and *Chop*. *18S* was selected as the most suitable gene for normalization. We measured the same ER stress marker genes to assess depot differences in ER stress when comparing LFD vs HFD in both BAT and VAT.

### For validation of Small-RNA-Seq data and to study depot differences in ER stress

We used 10 ng of RNA and TaqMan advanced miRNA cDNA synthesis kit (Applied Biosystems, Life Technologies, Carlsbad, CA) was used for poly (A) tailing, ligation, reverse transcription and miRNA amplification. For the final PCR reaction, we used TaqMan^®^ Advanced miRNA Assay (20X) of each miRNA tested and TaqMan^®^ Fast Advanced Master Mix (2X) (Applied Biosystems, Life Technologies, Carlsbad, CA). The miRNAs we tested were let-7a-5p, miR-30a-5p, miR-30c-2-3p, miR- 99b-5p, miR-143-3p, miR-199a-3p, miR-221-3p, miR-222-3p, miR-455-3p, and miR-708-5p. MiR-191-5p was used for normalization since this miRNA had similar expression in both adipose depots in the Small RNA sequencing. We measured the ER stress related miRNAs (miR-125b-5p, miR-143-3p, miR-222-3p, miR-30c-2-3p and miR-455-3p) to assess depot differences in ER stress when comparing LFD vs HFD in both BAT and VAT.

We analyzed the data from qPCR assays using 2- ΔΔCT method in the CFX Manager software (version 3.1) that was provided by Bio-Rad Laboratories, Inc. Two-groups were compared using Student’s *t*-tests using the above software with *p* < 0.05 considered as statistically significant.

## SUPPLEMENTARY MATERIALS FIGURES AND TABLES



## Abbreviations

ASPG: Asparaginase
ATF: Activating transcription factor
BAT: Brown adipose tissue
BCL2: B cell lymphoma 2
BIP: Heat shock protein family a (hsp70) member 5
CALR: Calreticulin
CASP2: Caspase
*CDKN1B*: Cyclin dependent kinase inhibitor 1B
CCL: CC-chemokine ligand
CHOP: CCAAT/Enhancer-Binding Protein Homologous Protein
CIDEA: Cell death-inducing DFFA-like effector A
ECHDC2: Enoyl-CoA hydratase domain containing 2
EIF2: Eukaryotic translation initiation factor 2
ELOVL: ELOVL fatty acid elongase
ER: Endoplasmic reticulum
FAM151A: Family with Sequence Similarity 151 Member A
GO: Gene Ontology
HFD: High fat diet
IL: Interleukin
IPA: Ingenuity^®^ Pathway Analysis
IRE1A: Inositol-requiring enzyme
LFD: Low fat diet
MCP1: Monocyte chemoattractant protein 1
MTOR: Mammalian target of rapamycin
MEV: Multiple Experiment Viewer
MIRNA/MiR: MicroRNA
MKK: Mitogen-activated protein kinase kinase
MMP: Matrix metalloproteinase
NRF2: Nuclear factor like 2
PERK: Protein kinase RNA-like ER kinase
PGC1A: PPARG coactivator 1 alpha
PPAR: Peroxisome proliferator activated receptor
PRDM: PR/SET domain
PTEN: Phosphatase and tensin
QPCR: Quantitative real time polymerase chain reaction
RNA-SEQ: RNA sequencing
RPKM: Reads per kilo base of exon model per million reads
RXR: Retinoid X receptor alpha
SAT: Subcutaneous adipose tissue
SERPINA: Serpin Family A Member
*SFRP*: Secreted frizzled-related protein
SHF: Src Homology 2 Domain Containing F
TNF: Tumor necrosis factor
TNFAIP8L1: Tumor necrosis factor alpha-induced *protein* 8-like *protein* 1
TWEAK: TNF related weak inducer of apoptosis
UCP1: Uncoupling protein 1
UPR: Unfolded protein response
VAT: Visceral adipose tissue
WAT: White adipose tissue
WFDC21: WAP four-disulfide core domain
XBP1: X-box binding protein-1

## References

[1] Ogden CL, Carroll MD, Kit BK, Flegal KM (2014). Prevalence of childhood and adult obesity in the United States, 2011-2012. JAMA.

[2] Guh DP, Zhang W, Bansback N, Amarsi Z, Birmingham CL, Anis AH (2009). The incidence of co-morbidities related to obesity and overweight: A systematic review and meta-analysis. BMC Public Health.

[3] de Ferranti S, Mozaffarian D (2008). The Perfect Storm: Obesity, adipocyte dysfunction, and metabolic consequences. Clinical Chemistry.

[4] Ouchi N, Parker JL, Lugus JJ, Walsh K (2011). Adipokines in inflammation and metabolic disease. Nat Rev Immunol.

[5] Bjorndal B, Burri L, Staalesen V, Skorve J, Berge RK (2011). Different adipose depots: their role in the development of metabolic syndrome and mitochondrial response to hypolipidemic agents. J Obes.

[6] Komolka K, Albrecht E, Wimmers K, Michal JJ, Maak S (2014). Molecular heterogeneities of adipose depots-potential effects on adipose-muscle cross-talk in humans, mice and farm animals. J Genomics.

[7] Harms M, Seale P (2013). Brown and beige fat: development, function and therapeutic potential. Nat Med.

[8] Shantikumar S, Caporali A, Emanueli C (2012). Role of microRNAs in diabetes and its cardiovascular complications. Cardiovasc Res.

[9] Williams MD, Mitchell GM (2012). MicroRNAs in insulin resistance and obesity. Exp Diabetes Res.

[10] Ge Q, Brichard S, Yi X, Li Q (2014). MicroRNAs as a new mechanism regulating adipose tissue inflammation in obesity and as a novel therapeutic strategy in the metabolic syndrome. J Immunol Res.

[11] Tripathi YB, Pandey V (2012). Obesity and endoplasmic reticulum (ER) stresses. Front Immunol.

[12] Kalupahana NS, Claycombe K, Newman SJ, Stewart T, Siriwardhana N, Matthan N, Lichtenstein AH, Moustaid-Moussa N (2010). Eicosapentaenoic acid prevents and reverses insulin resistance in high-fat diet-induced obese mice via modulation of adipose tissue inflammation. J Nutr.

[13] Ramsköld D, Wang ET, Burge CB, Sandberg R (2009). An abundance of ubiquitously expressed genes revealed by tissue transcriptome sequence data. PLOS Comput Biol.

[14] Pettersson US, Waldén TB, Carlsson PO, Jansson L, Phillipson M (2012). Female mice are protected against high-fat diet induced metabolic syndrome and increase the regulatory T cell population in adipose tissue. PLoS One.

[15] Mori H, Prestwich TC, Reid MA, Longo KA, Gerin I, Cawthorn WP, Susulic VS, Krishnan V, Greenfield A, Macdougald OA (2012). Secreted frizzled-related protein 5 suppresses adipocyte mitochondrial metabolism through WNT inhibition. J Clin Invest.

[16] Kalupahana NS, Voy BH, Saxton AM, Moustaid-Moussa N (2011). Energy-restricted high-fat diets only partially improve markers of systemic and adipose tissue inflammation. Obesity (Silver Spring).

[17] Sharp LZ, Shinoda K, Ohno H, Scheel DW, Tomoda E, Ruiz L, Hu H, Wang L, Pavlova Z, Gilsanz V, Kajimura S (2012). Human BAT possesses molecular signatures that resemble beige/brite cells. PLoS One.

[18] Wu Y, Smas CM (2008). Wdnm1-like, a new adipokine with a role in MMP-2 activation. Am J Physiol Endocrinol Metab.

[19] Lamming DW, Sabatini DM (2013). A central role for mTOR in lipid homeostasis. Cell Metabolism.

[20] Song MS, Salmena L, Pandolfi PP (2012). The functions and regulation of the PTEN tumour suppressor. Nat Rev Mol Cell Biol.

[21] Lv S, Ma M, Sun Y, Wang X, Qimuge N, Qin J, Pang W (2017). MicroRNA-129-5p inhibits 3T3-L1 preadipocyte proliferation by targeting G3BP1. Anim Cells Syst.

[22] Zhu L, Chen L, Shi CM, Xu GF, Xu LL, Zhu LL, Guo XR, Ni Y, Cui Y, Ji C (2014). MiR-335, an adipogenesis-related microRNA, is involved in adipose tissue inflammation. Cell Biochem Biophys.

[23] Deiuliis JA, Syed R, Duggineni D, Rutsky J, Rengasamy P, Zhang J, Huang K, Needleman B, Mikami D, Perry K, Hazey J, Rajagopalan S (2016). Visceral adipose microRNA 223 is upregulated in human and murine obesity and modulates the inflammatory phenotype of macrophages. PLoS One.

[24] Vohl MC, Sladek R, Robitaille J, Gurd S, Marceau P, Richard D, Hudson TJ, Tchernof A (2004). A survey of genes differentially expressed in subcutaneous and visceral adipose tissue in men. Obesity Research.

[25] Ma J, Jiang Z, He S, Liu Y, Chen L, Long K, Jin L, Jiang A, Zhu L, Wang J, Li M, Li X (2013). Intrinsic features in microRNA transcriptomes link porcine visceral rather than subcutaneous adipose tissues to metabolic risk. PLoS One.

[26] Wang T, Jiang A, Guo Y, Tan Y, Tang G, Mai M, Liu H, Xiao J, Li M, Li X (2013). Deep sequencing of the transcriptome reveals inflammatory features of porcine visceral adipose tissue. Int J Biol Sci.

[27] Gesta S, Tseng YH, Kahn CR (2007). Developmental origin of fat: tracking obesity to its source. Cell.

[28] Arias N, Aguirre L, Fernández-Quintela A, González M, Lasa A, Miranda J, Macarulla MT, Portillo MP (2016). MicroRNAs involved in the browning process of adipocytes. J Physiol Biochem.

[29] Duivenvoorde LP, van Schothorst EM, Bunschoten A, Keijer J (2011). Dietary restriction of mice on a high-fat diet induces substrate efficiency and improves metabolic health. J Mol Endocrinol.

[30] Wahli W, Michalik L (2012). PPARs at the crossroads of lipid signaling and inflammation. Trends Endocrinol Metab.

[31] Ortega-Molina A, Efeyan A, Lopez-Guadamillas E, Muñoz-Martin M, Gómez-López G, Cañamero M, Mulero F, Pastor J, Martinez S, Romanos E, Mar Gonzalez-Barroso M, Rial E, Valverde AM (2012). Pten positively regulates brown adipose function, energy expenditure, and longevity. Cell Metab.

[32] Bartoszewska S, Kochan K, Madanecki P, Piotrowski A, Ochocka R, Collawn JF, Bartoszewski R (2013). Regulation of the unfolded protein response by microRNAs. Cell Mol Biol Lett.

[33] Shimizu I, Walsh K (2015). The whitening of brown fat and its implications for weight management in obesity. Curr Obes Rep.

[34] Liu L, Yu X, Guo X, Tian Z, Su M, Long Y, Huang C, Zhou F, Liu M, Wu X, Wang X (2012). miR-143 is downregulated in cervical cancer and promotes apoptosis and inhibits tumor formation by targeting Bcl-2. Mol Med Rep.

[35] Dai R, Li J, Liu Y, Yan D, Chen S, Duan C, Liu X, He T, Li H (2010). MiR-221/222 suppression protects against endoplasmic reticulum stress-induced apoptosis via p27 (Kip1)- and MEK/ERK-mediated cell cycle regulation. Biological Chemistry.

[36] Andrews S (2010). FastQC: a quality control tool for high throughput sequence data. http://www.bioinformatics.babraham.ac.uk/projects/fastqc/.

[37] Creighton CJ, Reid JG, Gunaratne PH (2009). Expression profiling of microRNAs by deep sequencing. Brief Bioinform.

[38] Kim D, Pertea G, Trapnell C, Pimentel H, Kelley R, Salzberg SL (2013). TopHat2: accurate alignment of transcriptomes in the presence of insertions, deletions and gene fusions. Genome Biology.

[39] Kozomara A, Griffiths-Jones S (2014). miRBase: annotating high confidence microRNAs using deep sequencing data. Nucleic Acids Res.

[40] Creighton CJ, Nagaraja AK, Hanash SM, Matzuk MM, Gunaratne PH (2008). A bioinformatics tool for linking gene expression profiling results with public databases of microRNA target predictions. RNA.

[41] Saeed AI, Sharov V, White J, Li J, Liang W, Bhagabati N, Braisted J, Klapa M, Currier T, Thiagarajan M, Sturn A, Snuffin M, Rezantsev A (2003). TM4: a free, open-source system for microarray data management and analysis. BioTechniques.

[42] Oliveros JC (2007). VENNY. An interactive tool for comparing lists with Venn diagrams.

[43] Ashburner M, Ball CA, Blake JA, Botstein D, Butler H, Cherry JM, Davis AP, Dolinski K, Dwight SS, Eppig JT, Harris MA, Hill DP, Issel-Tarver L, and The Gene Ontology Consortium (2000). Gene ontology: tool for the unification of biology. Nat Genet.

[44] Abbas HA, Bui NH, Rajapakshe K, Wong J, Gunaratne P, Tsai KY, Coarfa C, Flores ER (2018). Distinct TP63 Isoform-Driven Transcriptional Signatures Predict Tumor Progression and Clinical Outcomes. Cancer Res.

[45] Venkatanarayan A, Raulji P, Norton W, Chakravarti D, Coarfa C, Su X, Sandur SK, Ramirez MS, Lee J, Kingsley CV, Sananikone EF, Rajapakshe K, Naff K (2015). IAPP-driven metabolic reprogramming induces regression of p53-deficient tumours *in vivo*. Nature.

