# Supplementary material for: An integrative transcriptomic approach to identify depot differences in genes and microRNAs in adipose tissues from high fat fed mice
Source: Oncotarget. 2018 Jan 13;9(10):9246–61. doi: 10.18632/oncotarget.24226 (PMC5823660; doi:10.18632/oncotarget.24226)
Supplement: Supplementary file 1 [file oncotarget-09-9246-s001.pdf]

An integrative transcriptomic approach to identify depot differences in genes and microRNAs in adipose tissues from high fat fed mice

SUPPLEMENTARY MATERIALS

Supplementary Table 1: Top ten differentially expressed genes in VAT/BAT comparisons of HFD fed mice

Name	BAT (RPKM)	VAT (RPKM))	Log2 fold change	*P* value
**Top upregulated genes in VAT (VAT/BAT comparison)**				
Gpnmb	1.40	510.99	8.51	4.38E–03
Ccl8	0.44	121.33	8.11	1.42E–02
Serpina3n	1.43	313.83	7.78	5.40E–03
Wfdc21	2.39	415.53	7.44	1.94E–03
Trem2	1.54	254.80	7.37	6.27E–03
Sfrp5	0.59	94.12	7.32	7.54E–03
Mmp3	0.40	54.98	7.11	6.00E–03
Ubd	4.34	594.54	7.10	1.65E–03
Mmp12	2.62	298.96	6.84	4.72E–03
Lgals3	18.88	2132.44	6.82	4.15E–03
**Top downregulated genes in VAT (VAT/BAT comparison)**				
**Name**	**BAT (RPKM)**	**VAT (RPKM))**	**Log2 fold change**	***P* value**
Ptgds	60.19	0.94	–6.00	1.64E–02
Aspg	110.70	4.26	–4.70	1.36E–02
Kng2	100.80	4.40	–4.52	1.13E–02
Lgr6	18.65	1.06	–4.13	1.17E–02
Slc27a2	84.48	5.73	–3.88	8.79E–03
Ntrk3	91.74	6.78	–3.76	9.62E–03
Cidea	27.38	2.27	–3.59	5.24E–03
Ucp1	5033.48	447.80	–3.49	8.21E–03
Fam151a	49.11	4.42	–3.47	1.41E–02
Cox7a1	2683.42	242.42	–3.47	1.20E–02

Abbreviations: Aspg (Asparaginase), Ccl8 (C-C Motif Chemokine Ligand 8), Cidea (Cell Death-Inducing DFFA-Like Effector A), Cox7a1 (Cytochrome C Oxidase Subunit 7A1), Fam151a (Family With Sequence Similarity 151 Member A), Gpnmb (Glycoprotein Nmb), Kng2 (Kininogen 2), Lgals3 (Lectin, Galactoside Binding Soluble 3), Lgr6 (Leucine-Rich Repeat Containing G Protein-Coupled Receptor 6), Mmp (Matrix Metallopeptidase), Ntrk3 (Neurotrophic Receptor Tyrosine Kinase 3), Ptgds (Prostaglandin D2 Synthase), Serpina3n (Serpin Family A Member 3), Sfrp5 (Secreted Frizzled-Related Protein 5), Slc27a2 (Solute Carrier Family 27 Member 2), Trem2 (Triggering Receptor Expressed On Myeloid Cells 2), Ubd (Ubiquitin D), Ucp1 (Uncoupling Protein 1), Wfdc21 (WAP four-disulfide core domain 21).

Supplementary Table 2: Top ten differentially expressed genes in SAT/BAT comparisons of HFD fed mice

Top upregulated genes in SAT (SAT/BAT comparison)				
**Name**	**BAT (RPKM)**	**SAT (RPKM))**	**Log2 fold change**	***P* value**
Sfrp5	0.59	137.43	7.87	8.55E–03
Ccl8	0.44	77.50	7.46	2.87E–02
Crtac1	0.47	72.38	7.28	1.52E–03
Mest	2.35	361.18	7.27	1.52E–03
Serpina3n	1.43	215.63	7.23	3.10E–03
Wfdc21	2.39	334.72	7.13	1.98E–03
Retn	30.92	3568.42	6.85	2.75E–03
Retnla	20.25	2319.68	6.84	1.01E–03
Lbp	2.47	248.82	6.65	4.11E–03
Mmp3	0.40	38.86	6.61	7.41E–03
**Top downregulated genes in SAT (SAT/BAT comparison)**				
**Name**	**BAT (RPKM)**	**SAT (RPKM))**	**Log2 fold change**	***P* value**
Ucp1	5033.48	5.87	–9.74	8.57E–03
Fabp3	2358.81	4.26	–9.11	3.46E–03
Aspg	110.70	0.87	–6.99	4.59E–03
Fam151a	49.11	0.76	–6.01	2.66E–03
Cox7a1	2683.42	45.38	–5.89	3.71E–03
Cpt1b	413.96	7.46	–5.79	1.75E–04
Acot11	30.14	0.60	–5.65	2.90E–03
Cidea	2710.06	54.39	–5.64	1.17E–03
Pank1	49.78	1.43	–5.12	2.17E–02
Me3	31.45	1.09	–4.85	1.25E–03

Abbreviations: Acot11 (Acyl-CoA Thioesterase 11), Aspg (Asparaginase), Ccl8 (C-C Motif Chemokine Ligand 8), Cidea (Cell Death-Inducing DFFA-Like Effector A), Cox7a1 (Cytochrome C Oxidase Subunit 7A1), Cpt1b (Carnitine Palmitoyltransferase 1B), Crtac1(Cartilage Acidic Protein 1), Fabp3 (Fatty Acid Binding Protein 3), Fam151a (Family With Sequence Similarity 151 Member A), Lbp (Lipopolysaccharide Binding Protein), Me3 (Malic Enzyme 3 Mest (Mesoderm Specific Transcript), Mmp3 (Matrix Metallopeptidase 3), Pank1 (Pantothenate Kinase1), Retn (Resistin), Retnla (Resistin Like Beta), Serpina3n (Serpin Family A Member 3), Sfrp5 (Secreted Frizzled-Related Protein 5), Ucp1 (Uncoupling Protein 1), Wfdc21 (WAP four-disulfide core domain 21),

Supplementary Table 3: Top differentially expressed genes in VAT/SAT comparisons of HFD fed mice

Top upregulated genes in VAT (VAT/SAT comparison)				
**Name**	**SAT (RPKM)**	**VAT (RPKM)**	**Log2 fold change**	***P* value**
Rgs1	3.33	35.17	3.40	3.54E–02
Smpd3	0.91	5.71	2.65	2.87E–02
Sash3	2.61	14.06	2.43	2.87E–02
Tbxas1	7.86	41.80	2.41	4.13E–02
Kcnn4	5.35	25.22	2.24	2.47E–02
Nfam1	3.99	18.29	2.20	3.50E–02
Cyth4	17.66	80.75	2.19	4.28E–02
Cd48	10.94	46.75	2.10	3.20E–02
Lrrc25	11.77	46.64	1.99	4.35E–02
Efhd2	28.10	104.92	1.90	2.44E–02

Top downregulated genes in VAT (VAT/SAT comparison)				
**Name**	**SAT (RPKM)**	**VAT (RPKM)**	**Log2 fold change**	***P* value**
Echdc2	54.00	22.73	–1.25	3.20E–02
Tnfaip8l1	10.12	4.58	–1.14	4.91E–02
Shf	10.25	5.12	–1.00	4.05E–02

Abbreviations:Cd48 (CD48 Molecule), Cyth4 (Cytohesin 4), Echdc2 (Enoyl–CoA Hydratase Domain Containing 2), Efhd2 (EF–Hand Domain Family Member D2), Kcnn4 (Potassium Calcium–Activated Channel Subfamily N Member 4), Lrrc25 (Leucine Rich Repeat Containing 25), Nfam1 (NFAT Activating Protein With ITAM Motif 1), Rgs1 (Regulator of G–Protein Signaling 1), Sash3 (SAM and SH3 Domain Containing 3), Shf (Src Homology 2 Domain Containing F), Smpd3 (Sphingomyelin Phosphodiesterase 3), Tbxas1 (Thromboxane A Synthase 1), Tnfaip8l1 (TNF Alpha Induced Protein 8 Like 1).

Supplementary Table 4: Top ten differentially expressed miRNAs with fold change values in SAT/BAT, VAT/BAT and VAT/SAT comparisons

Top up regulated miRNAs in pairwise comparisons of adipose depots					
**miRNA**	**SAT/BAT**	**miRNA**	**VAT/BAT**	**miRNA**	**VAT/SAT**
mmu–mir–196a–5p	7.41	mmu–mir–223–3p	6.76	mmu–mir–221–5p	3.49
mmu–mir–335–3p	6.31	mmu–mir–335–3p	5.7	mmu–mir–511–3p	2.79
mmu–mir–223–3p	5.83	mmu–mir–340–5p	4.92	mmu–mir–342–5p	2.59
mmu–mir–340–5p	5.65	mmu–mir–146b–5p	4.41	mmu–mir–194–5p	2.17
mmu–mir–298–5p	5.21	mmu–mir–708–5p	4.25	mmu–mir–130b–3p	2.08
mmu–mir–335–5p	5.13	mmu–mir–224–5p	4.19	mmu–mir–450a–5p	1.82
mmu–mir–708–5p	4.79	mmu–mir–298–5p	4.19	mmu–mir–1839–3p	1.77
mmu–mir–224–5p	4.62	mmu–mir–21a–5p	4.09	mmu–mir–22–3p	1.64
mmu–mir–196b–5p	4.52	mmu–mir–21a–3p	4.03	mmu–mir–222–3p	1.52
mmu–mir–152–5p	3.98	mmu–mir–142–5p	4.01	mmu–mir–146b–5p	1.52
**Top down regulated miRNAs in pairwise comparison of adipose depots**					
**miRNA**	**SAT/BAT`**	**miRNA**	**VAT/BAT**	**miRNA**	**VAT/SAT**
mmu–mir–378d	–6.8	mmu–mir–5105	–6.85	mmu–mir–6243	–3.0
mmu–mir–193b–5p	–6.33	mmu–mir–6243	–5.71	mmu–mir–335–5p	–2.46
mmu–mir–129–2–3p	–5.67	mmu–mir–193b–5p	–5.63	mmu–mir–5105	–2.3
mmu–mir–365–1–5p	–5.46	mmu–mir–378d	–5.51	mmu–mir–25–5p	–2.29
mmu–mir–129–5p	–5.38	mmu–mir–365–1–5p	–5.46	mmu–mir–3473b	–1.97
mmu–mir–122–5p	–5.23	mmu–mir–6240	–5.05	mmu–mir–3473e	–1.97
mmu–mir–6240	–5.15	mmu–mir–122–5p	–4.96	mmu–mir–5099	–1.79
mmu–mir–5130	–4.94	mmu–mir–455–3p	–4.7	mmu–mir–1843a–3p	–1.68
mmu–mir–1199–5p	–4.78	mmu–mir–129–5p	–4.6	mmu–mir–672–5p	–1.63
mmu–mir–2137	–4.72	mmu–mir–5130	–4.48	mmu–mir–19b–3p	–1.58

All the values in this table are log2 fold changes.

Supplementary Table 5: Top canonical pathways related to adipose tissues in the SAT/BAT comparison following a HFD

Activated canonical pathways in SAT relative to BAT	–log (*p*–value)	Ratio	z–score
EIF2 Signaling	19.900	0.500	7.550
Integrin Signaling	14.900	0.438	7.738
mTOR Signaling	14.500	0.447	4.899
Regulation of eIF4 and p70S6K Signaling	13.000	0.465	3.157
IL–8 Signaling	11.300	0.416	8.198
PI3K/AKT Signaling	10.100	0.460	3.159
Fcγ Receptor–mediated Phagocytosis in Macrophages and Monocytes	9.600	0.495	6.782
Paxillin Signaling	9.300	0.460	6.351
Rac Signaling	9.160	0.453	7.211
NGF Signaling	9.160	0.453	6.934
**Inhibited canonical pathways in VAT relative to BAT**	**–log (*p*–value)**	**Ratio**	**z–score**
PTEN Signaling	8.320	0.437	–4.429
RhoGDI Signaling	5.950	0.358	–5.082
Antioxidant Action of Vitamin C	5.430	0.398	–5.578
PPARα/RXRα Activation	4.510	0.331	–2.197
PPAR Signaling	2.400	0.323	–4.382

–log *p* value > 1.3 is equivalent to *p* value < 0.05 and is significant. Ratio is number of differentially expressed/total number of genes in a canonical pathway. *Z* score ≤ –2 is inhibited and ≥ 2 is activated.
Abbreviations: AKT (protein kinase B), EIF (Eukaryotic Translation Initiation Factor), mTOR (mechanistic target of rapamycin), IL8 (Interleukin–8), NGF (nerve growth factor), PI3K (phosphoinositide 3–kinase), *PTEN* (phosphatase and tensin homolog), PPAR (peroxisome proliferator activated receptor), RXR (Retinoid X receptor).

Supplementary Table 6: Top five canonical pathways related to adipose tissues in VAT/SAT comparison following a HFD

Canonical pathways	–log (*p*–value)	Ratio	No of mRNA
Rac Signaling	1.98	0.02	2/117
fMLP Signaling in Neutrophils	1.95	0.02	2/121
Prostanoid Biosynthesis	1.92	0.11	1/9
Leukocyte Extravasation Signaling	1.5	0.01	2/210
Signaling by Rho Family GTPases	1.37	0.01	2/247

–log *p* value > 1.3 is equivalent to *p* value < 0.05 and is significant. Ratio is number of differentially expressed/total number of genes in canonical pathway

Supplementary Table 7: Top molecular and cellular functions enriched by differentially expressed mRNA and miRNAs in VAT/SAT comparison in high fat fed mice

Molecular and cellular function	*p* value range	Number of mRNA	*p* value range	Number of miRNAs
VAT/SAT comparison				
Cellular development	4.66E–02 – 6.76E–06	9	4.02E–02 – 8.90E–08	22
Cellular growth and proliferation	4.47E–02 – 6.76E–06	8	3.52E–02 – 8.90E–08	22
Cell death and survival			4.26E–02 – 1.78E–04	18
Cellular movement	4.94E–02 – 1.36E–07	11	4.75E–02 – 9.00E–06	16

Cell cycle			4.26E–02 – 4.96E–05	11
Cell–to–cell signaling and interaction	4.57E–02 – 2.47E–05	9		
Cellular function and maintenance	4.56E–02 – 2.21E–04	15		

Note: We have mentioned only the top 5 molecular and cellular functions that were enriched by DEGs and differentially expressed miRNAs in IPA® analysis

Supplementary Table 8: Research diet: Composition of mineral and vitamin mix

	Content in HFD	Content in LFD
Fat, % energy	45	10
Carbohydrate, % energy	35	70
Protein, % energy	20	20
Digestible energy, kJ/g	19.8	16.1
	g/kg	
Lard	207	19
Soybean oil	29	24
Mineral mix,S100261	12	9
Dicalcium phosphate	15	12
Calcium carbonate	6	5
Potassium citrate, 1 H_2O	19	16
Vitamin mix, V100011	12	9
Choline bitartrate	2	2
dl–α–Tocopheryl acetate	0.15	0.12
	% total fatty acids	
Saturated fat	36.3	25.1
14:0	0.8	0.5
16:0	22.6	16.5
18:0	12.9	8.2
Monounsaturated fat	45.3	34.7
14:1	0.5	0.2
16:1 (*n*–9)	3.5	1.8
18:1 (*n*–9)	41.3	32.7
Polyunsaturated fat	18.5	40.2
18:2 (*n*–6)	14.9	34.6
18:3 (*n*–3)	1.9	5.0
20:4 (*n*–6)	1.6	0.7
20:5 (*n*–3)	0.0	0.0

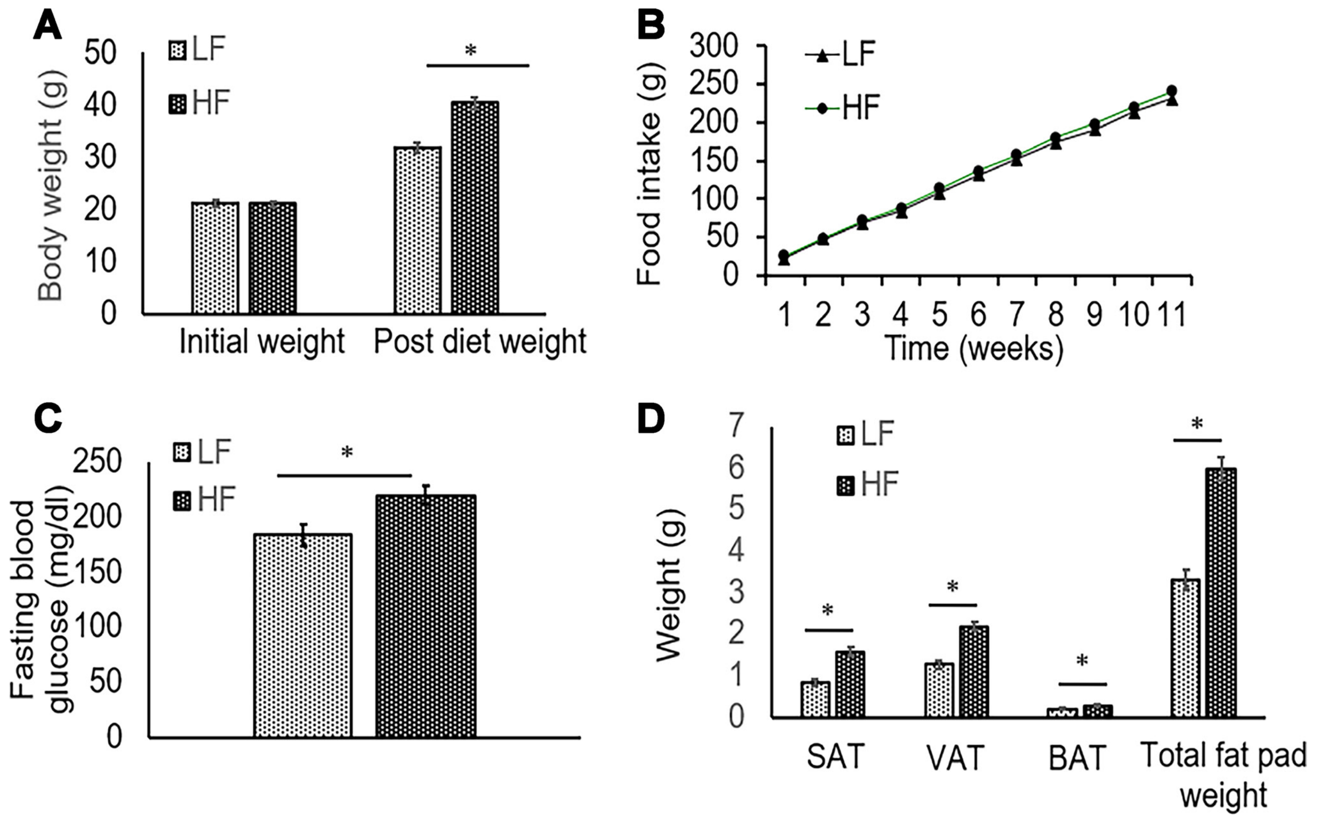

Supplementary Figure 1: Differences in body weight, fasting blood glucose, total fat and adipose depots weights between low fat (LF) and high fat (HF) fed mice (*n* = 10 per group). (**A**) Starting weight at 5–6 weeks aged mice and after 11 weeks of high and low fat diets. (**B**) Food intake during the 11 week intervention. (**C**) Fasting blood glucose after 11 weeks of high and low fat diets. (**D**) Differences in total fat pad weights and various adipose depot weights after 11 weeks of high and low fat diets.

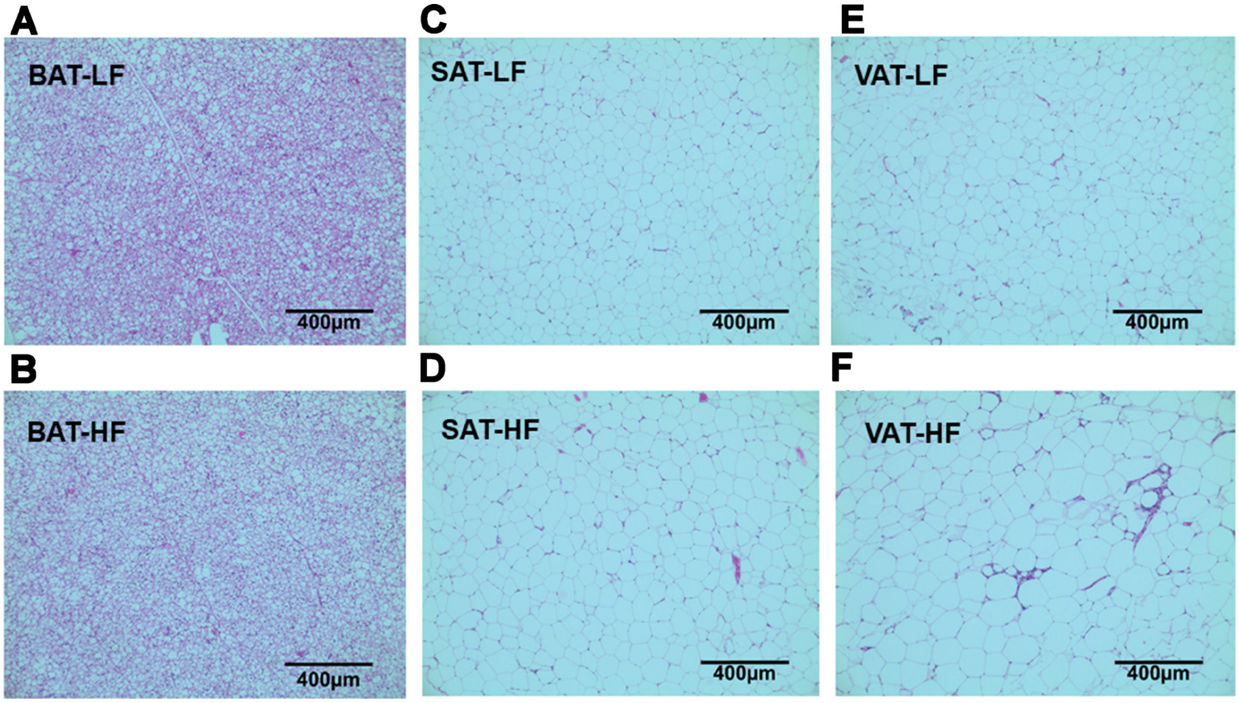

Supplementary Figure 2: Representative hematoxylin and eosin stained histological illustration of adipose tissue from different depots of high fat (HF) and low fat (LF) fed mice as demonstrated by light microscopy. Adipocyte size differences are observed between. low fat and high fat fed mice at 10X magnification. (**A**) BAT-LF, (**B**) BAT-HF, (**C**) SAT-LF, (**D**) SAT-HF, (**E**) VAT-LF, (**F**) VAT-HF.

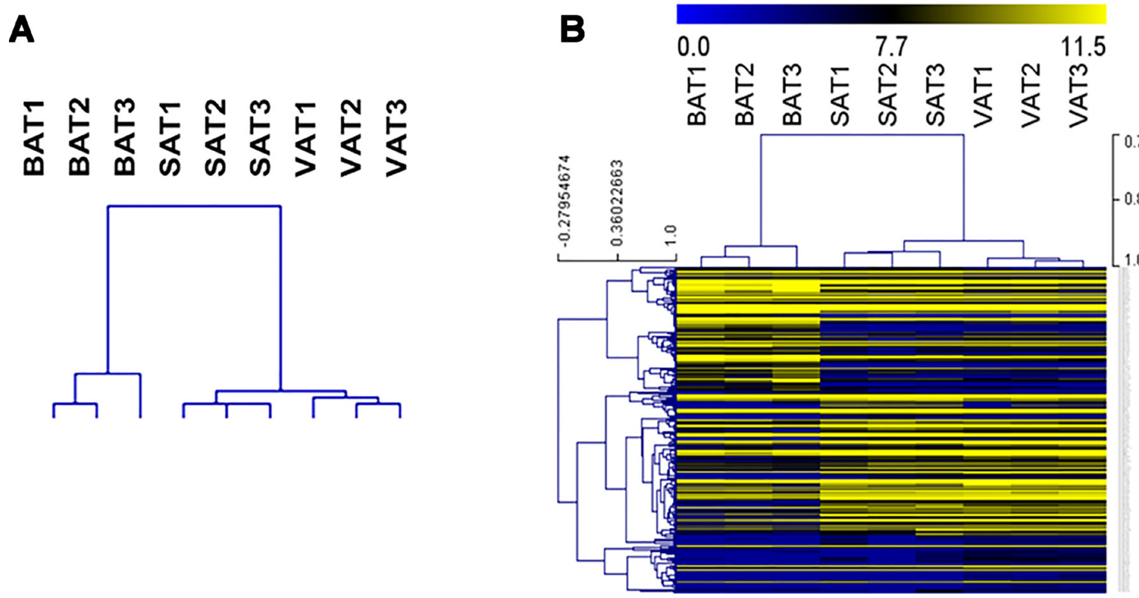

Supplementary Figure 3: Gene-miRNA expression patterns between adipose depots of high fat fed mice (**A**) Dendrogram of expressed genes 14,213 genes expressed in adipose tissues (BAT, SAT and VAT) (with RPKM ≥ 0.3 in depots) and (**B**) Hierarchical clustering of miRNAs for the 246 differentially expressed miRNAs expressed in BAT, SAT and VAT from HFD fed mice

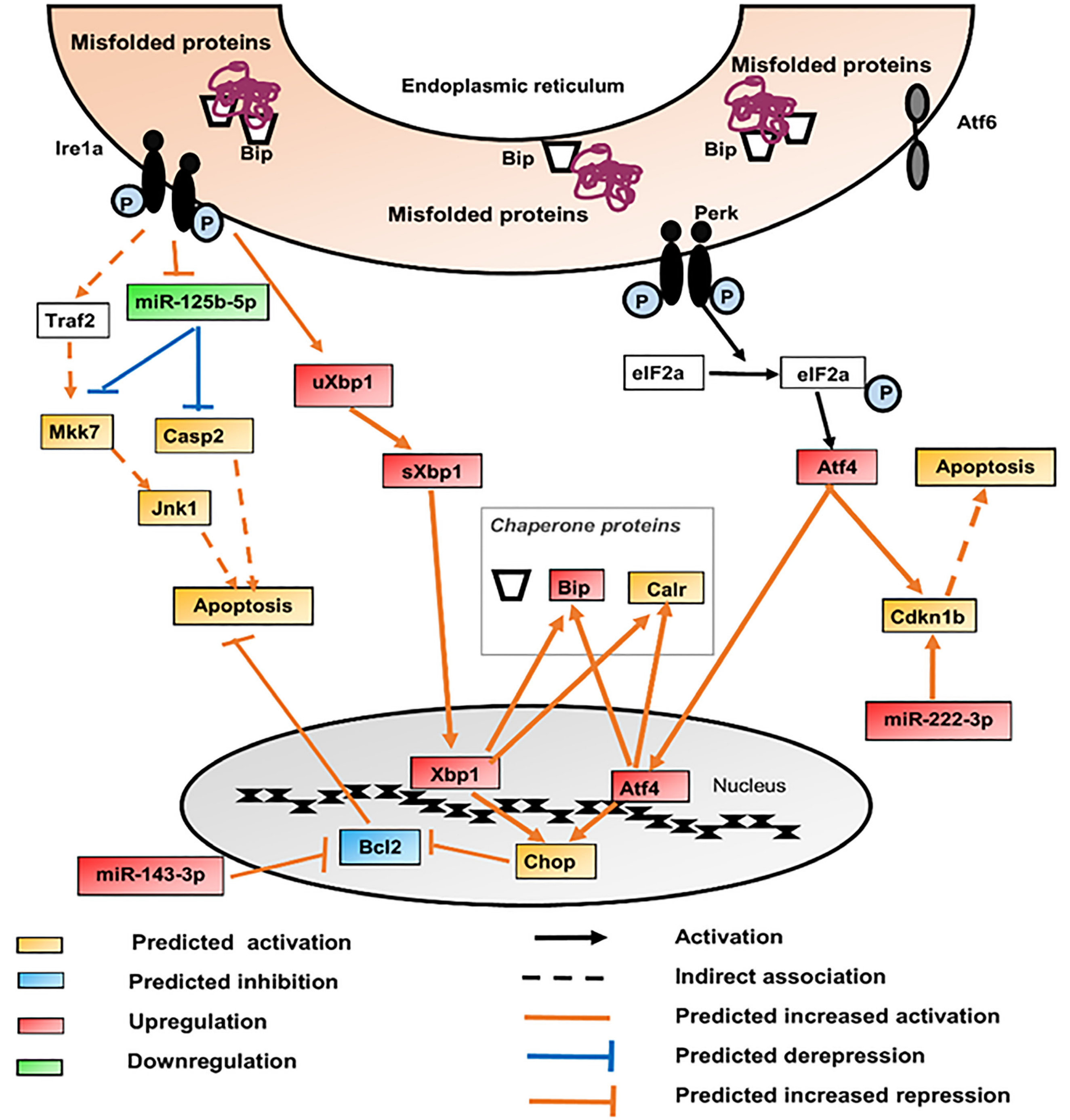

Supplementary Figure 4: miRNA-mRNA associations related to ER stress that are altered in VAT by HFD in mice. Upregulated and down regulated genes and miRNAs in VAT-HF vs VAT-LF are based on qPCR. The predicted activation and inhibition of genes and processes in VAT-HF/VAT-LF comparison are based on the prediction from IPA® program based on existing literature. miRNA and mRNA activation and inhibitions are predicted also based on past literature. Abbreviations: Bcl2 (B cell lymphoma 2), Calr (calreticulin), Casp2 (caspase 2), Cdkn1b (Cyclin Dependent Kinase Inhibitor 1B), eiF2α (Eukaryotic Translation Initiation Factor 2A), JNK (C-Jun N-terminal kinase), Mkk7 (mitogen-activated protein kinase kinase 7), Traf2 (TNF receptor-associated factor 2). uXBP1 (unspliced XBP1).

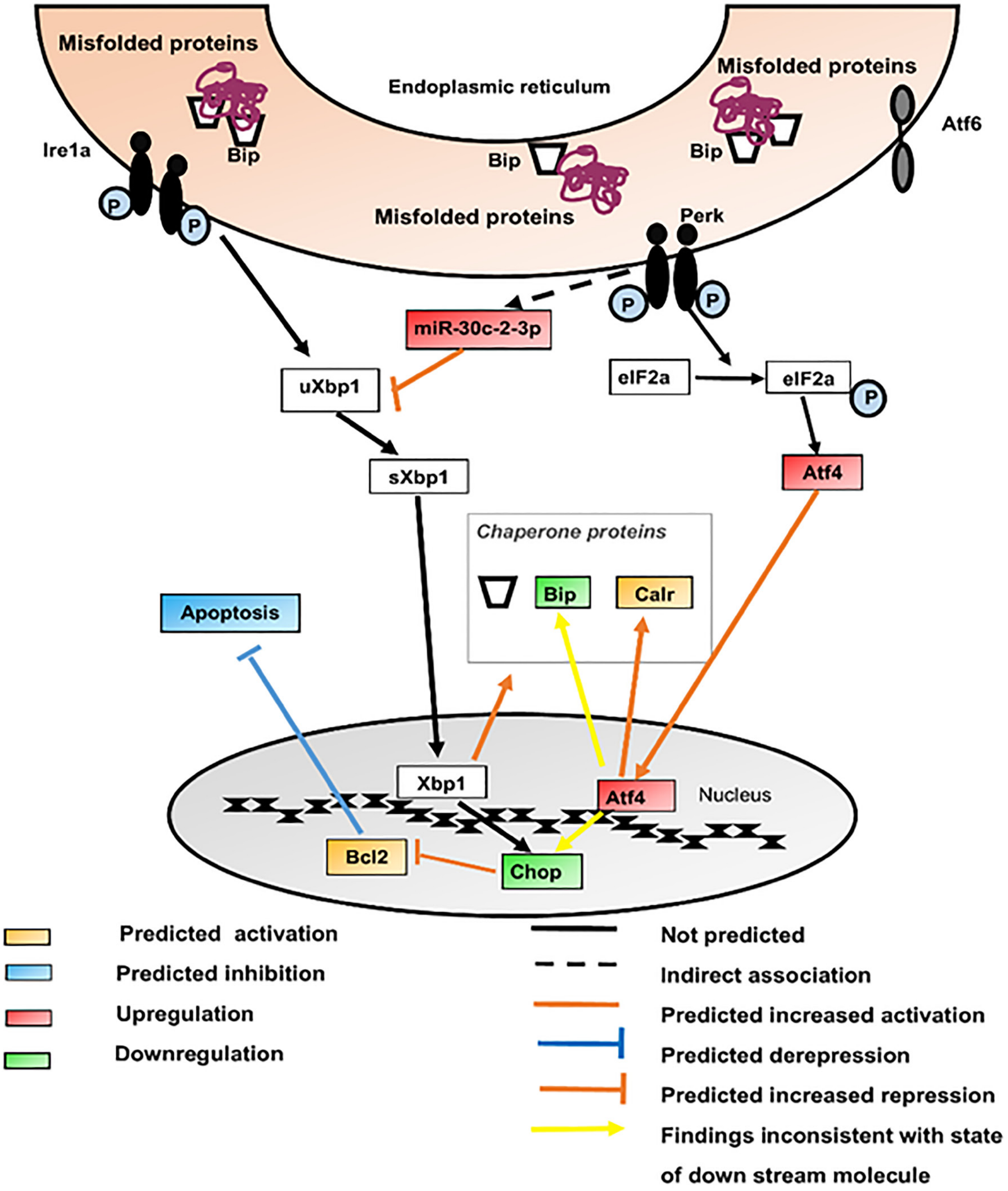

Supplementary Figure 5: miRNA–mRNA associations related to ER stress that are altered in BAT by HFD in mice. Upregulated and down regulated genes and miRNAs in BAT–HF vs BAT–LF are based on qPCR. The predicted activation and inhibition of genes and processes in BAT–HF/BAT–LF comparison are based on the prediction from IPA® program based on existing literature. miRNA and mRNA activation and inhibitions are predicted also based on past literature. Abbreviations: Bcl2 (B cell lymphoma 2), Calr (calreticulin), eiF2α (Eukaryotic Translation Initiation Factor 2A), uXBP1 (unspliced XBP1).

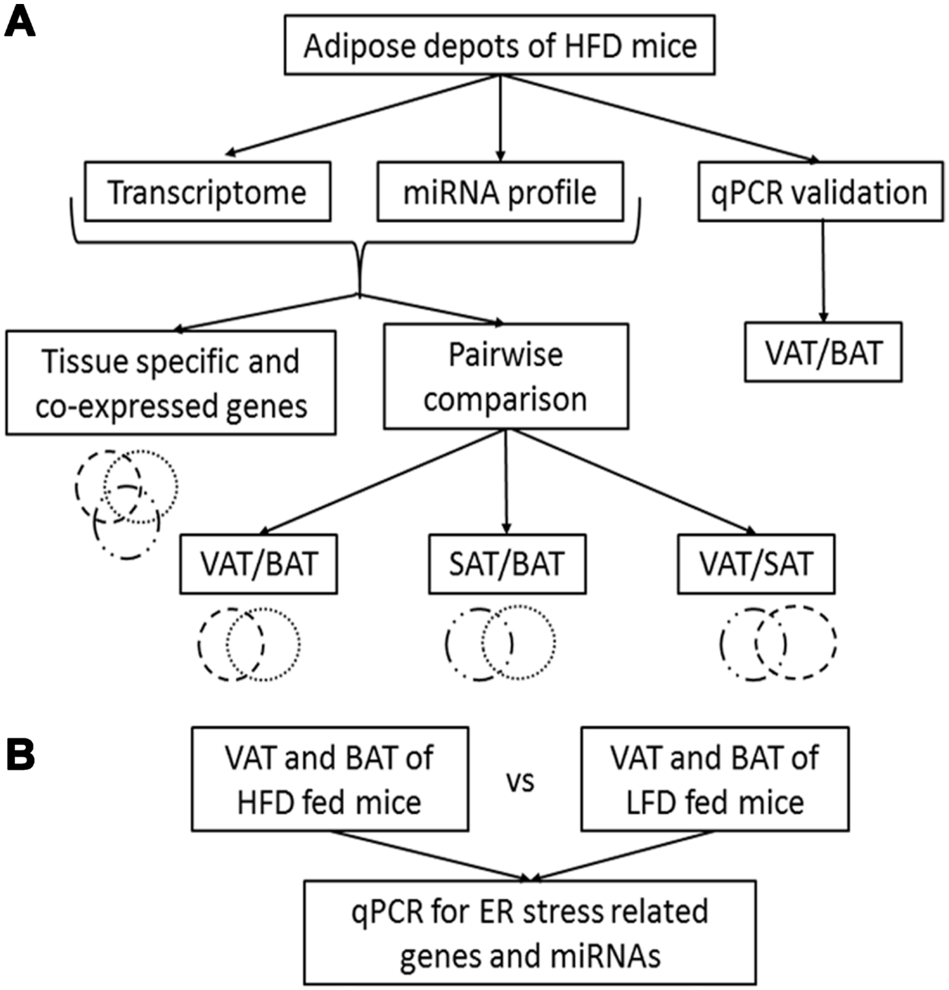

Supplementary Figure 6: Study design. Male C57BL/6J mice 6–8 weeks of age were fed a high–fat diet (HFD) or low fat diet (LFD) for 11 weeks. (**A**) Global RNA sequencing for whole transcriptome and miRNA profiles in BAT, SAT and VAT from HFD fed mice was performed; tissue specific, co–expressed, differentially expressed genes and miRNAs were identified in the 3 adipose tissue depots. (**B**) LFD vs HFD comparison was performed for VAT and BAT separately for ER stress marker genes.

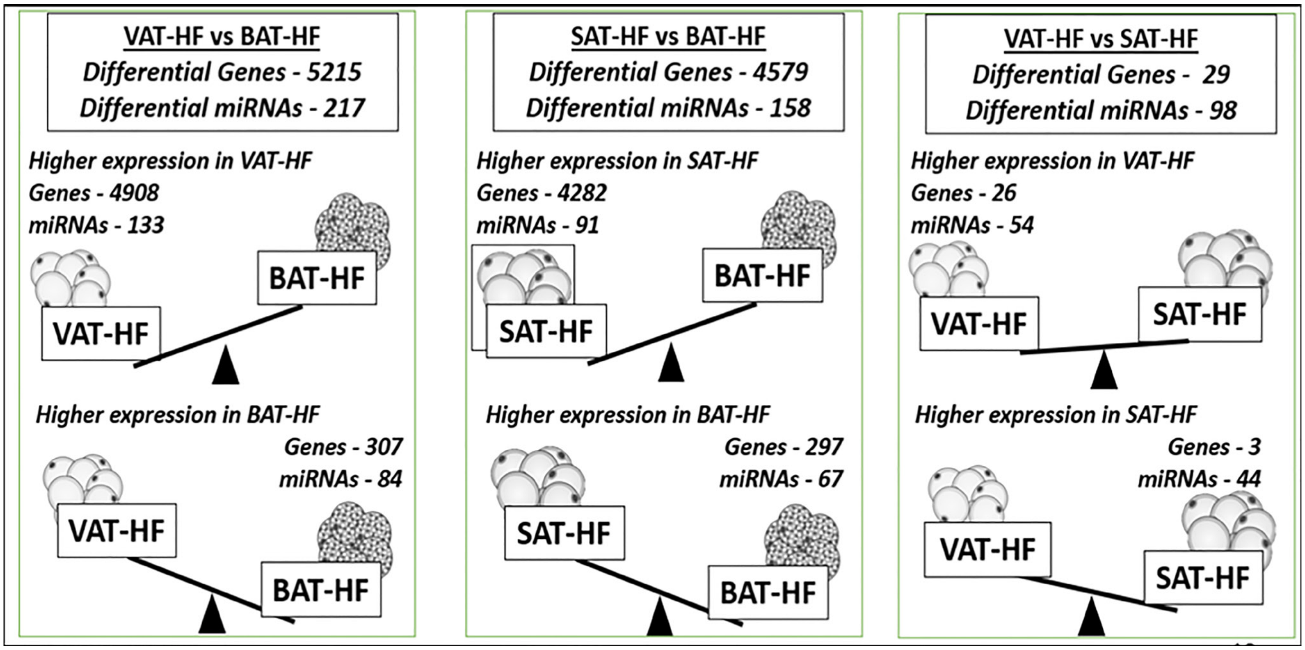

Supplementary Figure 7: Differentially expressed genes (DEG) and miRNAs (DE miRNA) in pairwise comparison of adipose depots in HFD fed mice subjected to functional analysis using Ingenuity Pathway Analysis software. We used differentially expressed genes with RPKM of 1 or more in both tissues being compared with 2 or more-fold change with 95% confidence. The differentially expressed miRNAs had fold change of 1.25 or more with 95% confidence. Genes and miRNA with higher expression are indicated by taller columns for each pairwise comparison.
